# Profiling of drugs and environmental chemicals for functional impairment of neural crest migration in a novel stem cell-based test battery

**DOI:** 10.1007/s00204-014-1231-9

**Published:** 2014-04-02

**Authors:** B. Zimmer, G. Pallocca, N. Dreser, S. Foerster, T. Waldmann, J. Westerhout, S. Julien, K. H. Krause, C. van Thriel, J. G. Hengstler, A. Sachinidis, S. Bosgra, M. Leist

**Affiliations:** 1 Center for Stem Cell Biology, Sloan-Kettering Institute for Cancer Research, New York City, NY USA; 2 Developmental Biology Program, Sloan-Kettering Institute, New York City, NY USA; 3 Department of Biology, University of Konstanz, 78457 Constance, Germany; 4 Nederlandse Organisatie voor Toegepast Natuurwetenschappelijk Onderzoek (TNO), 2628 VK Delft, The Netherlands; 5 Department of Pathology and Immunology, Geneva Medical Faculty, University of Geneva, 1211 Geneva, Switzerland; 6 Center of Physiology and Pathophysiology, Institute of Neurophysiology, University of Cologne, 50931 Cologne, Germany; 7 Leibniz Research Centre for Working Environment and Human Factors (IfADo), Technical University of Dortmund, 44139 Dortmund, Germany; 8 Doerenkamp-Zbinden Chair for In Vitro Toxicology and Biomedicine, University of Konstanz, Box 657, Universitätsstr. 10, 78457 Constance, Germany

**Keywords:** Test battery-based compound screening, Developmental toxicity testing, hESC-based test system, Neural crest migration assay

## Abstract

Developmental toxicity in vitro assays have hitherto been established as stand-alone systems, based on a limited number of toxicants. Within the embryonic stem cell-based novel alternative tests project, we developed a test battery framework that allows inclusion of any developmental toxicity assay and that explores the responses of such test systems to a wide range of drug-like compounds. We selected 28 compounds, including several biologics (e.g., erythropoietin), classical pharmaceuticals (e.g., roflumilast) and also six environmental toxicants. The chemical, toxicological and clinical data of this screen library were compiled. In order to determine a non-cytotoxic concentration range, cytotoxicity data were obtained for all compounds from HEK293 cells and from murine embryonic stem cells. Moreover, an estimate of relevant exposures was provided by literature data mining. To evaluate feasibility of the suggested test framework, we selected a well-characterized assay that evaluates ‘migration inhibition of neural crest cells.’ Screening at the highest non-cytotoxic concentration resulted in 11 hits (e.g., geldanamycin, abiraterone, gefitinib, chlorpromazine, cyproconazole, arsenite). These were confirmed in concentration–response studies. Subsequent pharmacokinetic modeling indicated that triadimefon exerted its effects at concentrations relevant to the in vivo situation, and also interferon-β and polybrominated diphenyl ether showed effects within the same order of magnitude of concentrations that may be reached in humans. In conclusion, the test battery framework can identify compounds that disturb processes relevant for human development and therefore may represent developmental toxicants. The open structure of the strategy allows rich information to be generated on both the underlying library, and on any contributing assay.

**Electronic supplementary material:**

The online version of this article (doi:10.1007/s00204-014-1231-9) contains supplementary material, which is available to authorized users.

## Introduction

Individual human embryonic stem cell-based developmental toxicity test systems have been established by several laboratories (Jagtap et al. 2011; Balmer et al. 2012; Stummann et al. 2009). A next step will be the combination of these and other assays to a comprehensive battery able to predict human developmental toxicities (Leist et al. 2012b; van Thriel et al. 2012). Cultures of differentiating pluripotent stem cells such as human embryonic stem cells (hESC) or human-induced pluripotent stem cells (Leist et al. 2008a; Thomson et al. 1998; Takahashi et al. 2007) offer unique possibilities of studying the very early steps of human development that lead to the formation of germ layers and primordial tissues. This opportunity was seized by the European Union research consortium for the use of ‘embryonic stem cell-based novel alternative tests’ (ESNATS) for the prediction of toxicity of drug candidates (www.esnats.eu). This project focused on the one hand on transcriptomics-based toxicity predictions (Krug et al. 2013b; Kuegler et al. 2010). On the other hand, several tests were established, which allowed the assessment of neurochemical and cell biological cell functions (Stiegler et al. 2011; Zimmer et al. 2011b; 2012; Krug et al. 2013a) and of complex cell interactions (Preynat-Seauve et al. 2009; Kuegler et al. 2012). Moreover, concepts have been developed to compare relevant in vitro and in vivo concentrations (Bosgra et al. 2012; Krug et al. 2013a; Zimmer et al. 2011a), and to incorporate systems for metabolic activation of drugs (Godoy et al. 2013). It is assumed by many experts that the combination of such different tests in a battery may eventually be able to predict human developmental toxicity (Basketter et al. 2012; Piersma et al. 2013; Schenk et al. 2010). The hESC-based test systems of ESNATS cover different aspects of development. For instance, the UKK system (Meganathan et al. 2012) models early multi-germ-layer differentiation, while the UKN1 system (Balmer et al. 2012) models specific neuroectodermal differentiation. The UKN2 system, also known as ‘migration inhibition of neural crest’ assay (MINC) (Zimmer et al. 2012) is a functional test probing the inhibition of neural crest cell migration by chemicals. During the initial establishment of the assays, only a small number of positive and negative controls were tested. Therefore, the applicability domain of these assays and their response dynamics when faced with a broader variety of compounds are unknown. Moreover, the information from only few compounds is not sufficient to evaluate how far the test systems are complementary, and where they may be redundant in the information they provide.

In DNT test library selection, new approaches are required (Leist et al. 2012a) to break a vicious circle between lack of sufficient tool compounds, and the inability to classically validate test systems without such compounds (Leist et al. 2010, 2012b). One of these would be a screening approach of hitherto little characterized compounds in multiple test systems. This would provide information on which biological processes may be targeted by the compounds. Together with mechanistic studies on the mode of action, this approach may allow to build a case for a hazard estimate independent of correlations with in vivo data (Kadereit et al. 2012). Moreover, characterization of the available assays would be promoted.

For the design of such a battery of different tests, experience from earlier approaches can be used as guidance. Test batteries may for instance be constructed in a tiered way to avoid redundant testing. If information on each compound from every test is desired, then non-tiered approaches are more useful. Examples from the field of reproductive toxicity testing are for instance the ReProTect feasibility study (Schenk et al. 2010) or the ChemScreen test battery (Piersma et al. 2013). Non-tiered testing is also performed in the ToxCast Program, in which hundreds of tests have been run in parallel, to use the data afterward—in combination with pre-existing in vivo data—for predictions of drivers and mechanisms of reproductive toxicity (Kleinstreuer et al. 2011; Padilla et al. 2012; Sipes et al. 2011).

Here, we defined a framework for a test battery, and we provided an initial characterization of a core set of test compounds which can be expanded at later stages. To evaluate the feasibility of the suggested framework and the usefulness of the set of compounds, we selected one well-characterized assay for a first screen. The MINC assay (Zimmer et al. 2012) was selected, as it is based on a functional endpoint, and it affords sufficient throughput to evaluate a compound battery of that size. The underlying biological rationale of the test is that disturbance of neural crest migration by toxicants leads to severe malformations in different species. Several factors (e.g., genetics and chemicals) have already been identified as causes for neural crest (NC)-related developmental defects (Di Renzo et al. 2007; Fuller et al. 2002; Menegola et al. 2000). Identification of several hits in such a functional assay provides a good starting point for future characterization of the compounds by more phenotypic assays and for correlations of functional disturbances with, e.g., transcriptome changes.

## Materials and methods

### Cell culture

The reporter hES cell line H9-Dll1 (GFP under Dll1 promoter) was provided by Mark Tomishima from the Memorial Sloan-Kettering Cancer Centre (MSKCC, NY, USA). Import of the cells and all experiments were carried out according to German legislation under the license number 1710-79-1-4-27 of the Robert-Koch Institute. H9-Dll1 cells were maintained on Mouse Embryonic Fibroblasts (MEFs) in DMEM/F12 (Gibco) medium containing 20 % of serum replacement, HEPES (1 M, Gibco), l-glutamine (Glutamax, Gibco), non-essential amino acids (MEM NEAA, Gibco), beta-mercaptoethanol (Gibco) and basic fibroblast growth factor (10 ng/ml, Invitrogen). The murine ES cell line CGR8 was obtained from the European Collection of Cell Culture (ECACC, UK). CGR8 cells were maintained on 0.1 % gelatin-coated dishes in BHK21 medium, supplemented with 10 % fetal calf serum, l-glutamine, non-essential amino acids, penicillin/streptomycin and leukemia inhibitory factor (Kern et al. 2013). HEK293 (CRL-1573, ATCC) cell line was maintained in DMEM supplemented with 10 % fetal calf serum at 37 °C in a humidified atmosphere containing 5 % CO_2_.

### Neural differentiation protocols

The mESC cell line (CGR8) was differentiated toward a neural stem cell phenotype using the protocol described by Barberi et al. (2003). Briefly, CGR8 were seeded on irradiated MS5 cells and cultivated in DMEM medium containing 15 % Knock-out Serum Replacement, non-essential amino acids, beta-mercaptoethanol and penicillin/streptomycin. After 4 days, cells were replated on polyornithine (15 μg/ml)-coated dishes in N2 medium containing DMEM, N2 supplement, penicillin/streptomycin and 10 ng/ml of basic human fibroblast growth factor (Invitrogen). Differentiation of hESC into neural crest cells was initiated on Mitomycin C-treated murine bone marrow-derived stromal MS5 cell line and continued as described in Zimmer et al. (2012).

### Evaluation of a non-cytotoxic range by resazurin assay and benchmark concentration (BMC) calculation

The effects of the toxic compounds on cell viability of two cell lines were evaluated by using the resazurin assay. The assay is based on the capability of viable and healthy cells to reduce resazurin to resorufin, which can be measured by a colorimetric or fluorimetric shift as described earlier (Zimmer et al. 2012). HEK293 cells and mESC-derived neural stem cells (mESCn) were exposed for 48 h to the different substances. mESCn were exposed to test compounds after 6 days of differentiation. After this period, the cells were incubated at 37 °C and 5 % CO_2_ with 10 μg/ml resazurin for 30 min (HEK293) or up to 5 h (neural stem cells). The background fluorescence of resazurin itself was determined by including a resazurin only control. Resazurin reduction was analyzed in cell culture medium fluorimetrically (*λ*_ex_ = 530 nm, *λ*_em_ = 590 nm). These data were used to model a concentration–response curve and to calculate the concentration corresponding to a 10 % reduction in viability (BMC10). In addition, the BMC15 and the lower limit of its 95 % CI (BMCL15) were determined. This latter value was used as estimate for the upper boundary of the non-cytotoxic concentration range.

### Cell migration analysis

Cell migration analysis was carried out using a scratch assay design as described in Lee et al. (2009) and Zimmer et al. (2012) with minor modifications. hESC-derived NCCs were grown to a confluent monolayer using 48-well plates (Corning). Right before starting the assay, each well was scratched using a 20-μl pipette tip in order to create a cell-free gap. The medium was removed and replaced by fresh medium containing the test chemicals. The width of the cell-free gap was determined right after scratching in a control plate in order to define the dimension of the region of interest (ROI) for the analysis. The cells were exposed to the toxicants for 48 h; after this period, the general cytotoxicity was assessed by the resazurin reduction assay. Migration of NCC was evaluated by florescence microscopy analysis. In order to easily count the number of cells, incubation with fresh medium containing the DNA dye H-33342 (1 μg/ml) was performed for 30 min. After the incubation period, random images along the scratch were taken at 4× magnification. The number of cells with H-33342-positive nuclei within the ROI was automatically calculated by the use of a KNIME flowchart.

### Chemical exposure during migration

hESC-derived neural crest cells were exposed to chemicals in N2 medium containing EGF (20 ng/ml) and FGF2 (20 ng/ml). For a detailed list of chemicals and their tested concentration range used in this study, see Fig. 3 and Fig. S1, S2.

### In vitro: in vivo comparison of toxicity data by PBPK modeling

In order to evaluate the clinical relevance of the in vitro concentrations found to impair the migration of the hESC-derived NCCs in this study, a three-step (physiology-based) pharmacokinetic (PBPK) modeling strategy has been used, as already described in Krug et al. (2013b) and Piersma et al. (2013). Briefly, the following steps were taken: (a) choice of an appropriate absorption, distribution, metabolism, excretion (ADME) model; (b) use of this model to simulate plasma and/or target tissue concentrations in time corresponding to the exposure (dose, route of administration, interval) at which relevant toxic effects were observed in already published in vivo studies; (c) calculation of the nominal concentration in vitro that has the same unbound concentration as the toxic concentration in vivo (when possible).

#### In vitro: in vivo comparison of toxicity data for interferon β

A PBPK model for the analysis of interferon β (IFN-β) kinetics in monkeys, described by Mager et al. (2003), was implemented in the acslX software (version 3.0.2.1, Aegis Technologies) (step a). The original model was built on the basis of data from 18 cynomolgus monkeys that were exposed i.v. to single doses of 1, 3, 10 MIU/kg and then to a s.c dose of 0.3 ml/kg of IFN-β. *In vivo* developmental toxicity concentrations of the drug have been extrapolated from a study reporting the effects of the exposure of IFN-β in pregnant cynomolgus monkeys (http://www.fda.gov/downloads/Drugs/DevelopmentApprovalProcess/HowDrugsareDevelopedandApproved/ApprovalApplications/TherapeuticBiologicApplications/ucm106138.pdf) (step b)

#### In vitro: in vivo comparison of toxicity data for triadimefon

A PBPK model for the pesticide triadimefon and its metabolite triadimenol in rats published by Crowell et al. (2011) was reconstructed in acslX and used to predict the target tissue concentration related to the exposure scenarios leading to toxic effects on male fertility and CNS toxicity (step a). Developmental toxicity-inducing concentrations were extrapolated from the in vivo study by Goetz et al. (2007), in which pregnant rats have been exposed to the pesticide. Two exposure scenarios were simulated: Dietary exposure assuming a constant intake of the entire drug dose within the first 12 h of 24-h periods; oral gavage, modeled as a bolus dose into the liver compartment (step b). The nominal in vitro concentrations equivalent to the concentrations predicted in vivo were determined correc ting for the differences in albumin concentration and lipid fraction between plasma or cerebrospinal fluid and test medium, using the follow equations:

$$\begin{gathered} EC_{x} = EC_{p} \times \left\{ {(1 - f_{b,p} ) \times \frac{{1 + K_{ow} \times VF_{L,x} }}{{1 + K_{ow} \times VF_{L,p} }} + f_{b,p} \times \frac{{P_{x} }}{{P_{p} }}} \right\} \hfill \\ EC_{u,x} = \frac{{EC_{x} - \frac{{P_{x} }}{{P_{p} }} \times f_{b,p} \times EC_{p} }}{{1 + K_{ow} \times VF_{L,x} }} \hfill \\ \end{gathered}$$

where EC represents the effective concentration; *f*_*b,p*_ the plasma fraction unbound; *K*_*ow*_ the octanol:water partition coefficient; VFL the lipid fraction; *P* the albumin concentration; suffix *u* means unbound; suffix *p* the plasma; and suffix *x* the other medium (in vitro or CSF) (step c).

The parameters of free (unbound) fraction, octanol:water partition and blood:plasma concentration ratio were taken from the published study by (US EPA 2006) and 0.11, 912 and 0.84, respectively. Data for rat CSF (estimated as 0.5 % of plasma) were taken from (Habgood et al. 1992; Koch et al. 2001) and data for MINC culture medium were calculated based on information provided by the supplier.

#### In vitro: in vivo comparison of toxicity data for PBDE-99

A PBPK model was constructed based on data of tissue distribution, metabolism and excretion of PBDE-99 as described by Hakk et al. (2002) and Chen et al. (2006) (step a). The PBPK model structure to describe the kinetics of PBDE-99 is shown in Fig. S4a. The model contains a gastrointestinal lumen compartment (GI), two rapid equilibrium compartments (T1 and T2), a blood compartment (B), a lipophilic tissues compartment (F) representing adipose tissue and skin, and compartments for urinary and fecal excretion (Ur and Fe). The exchange between blood and tissue compartments is described by first-order rate constants k_b1_, k_1b_, k_b2_, k_2b_, k_bf_ and k_fb_ with unit h^−1^. The compound is absorbed into T1—containing intestinal tissues and liver, but not further specified—by a rate k_ab_, and eliminated back into GI with rate k_el_. Excretion occurs from the blood compartment with rate k_ur_ and from the GI compartment with rate k_fe_. The model was described as a set of differential equations in acslX. Concentrations were calculated from amounts by dividing compartment volumes: 0.21, 0.56 and 0.06 ml/g BW (body weight) for lipophilic tissues, rapid equilibrium tissues and blood, respectively, as reported by Brown et al. (1997). The estimated parameter values are listed in the supplemental material (Fig. S3b). The model performance was demonstrated by comparison of model predictions to in vivo PK data reported by Chen et al. (2006) for a single oral dose of 1 μmol/kg (Fig. S3 c,d) and an intravenous bolus dose of 1 μmol/kg (Fig. S3e, f). *In vivo* developmental toxicity concentrations were extrapolated by the data from Kuriyama et al. (2005) and Viberg et al. (2005), where neuro-developmental effects are observed in rats exposed to PBDE-99, during the gestational or the early infancy period (step b).

### Statistics and data mining

For the resazurin assay, five technical replicates for HEK293 cells and four biological replicates for mESCn cells have been analyzed for each compound and concentration. For the migration assay, the number of migrated cells was automatically counted in ≥4 different images per experiment by a KNIME flowchart-based software. All data displayed are means from three independent biological experiments. Each biological experiment consisted of at least four technical replicates. Statistical differences were tested with GraphPad Prism 5.0 (Graphpad Software, La Jolla, USA) by applying ANOVA using Bonferroni’s post hoc test. Independent biological experiments (not technical replicates) were the basic unit used for statistical testing.

## Results

### Considerations and design principles of the test battery

Several murine and human stem cell-based developmental toxicity test systems have been developed by ESNATS project partners (Balmer et al. 2012; Krug et al. 2013b; Stiegler et al. 2011; Zimmer et al. 2012; Kern et al. 2013; Jagtap et al. 2011) and others (Fritsche et al. 2011; Seiler and Spielmann 2011; Hogberg et al. 2010; Pallocca et al. 2013; Piersma et al. 2013; Suzuki et al. 2011). All these assays have been evaluated individually with positive and negative control compounds regarding their biological relevance for fundamental processes of mammalian development. However, little is known how such test systems can be combined to yield information on drug toxicity.

**Fig. 1:**
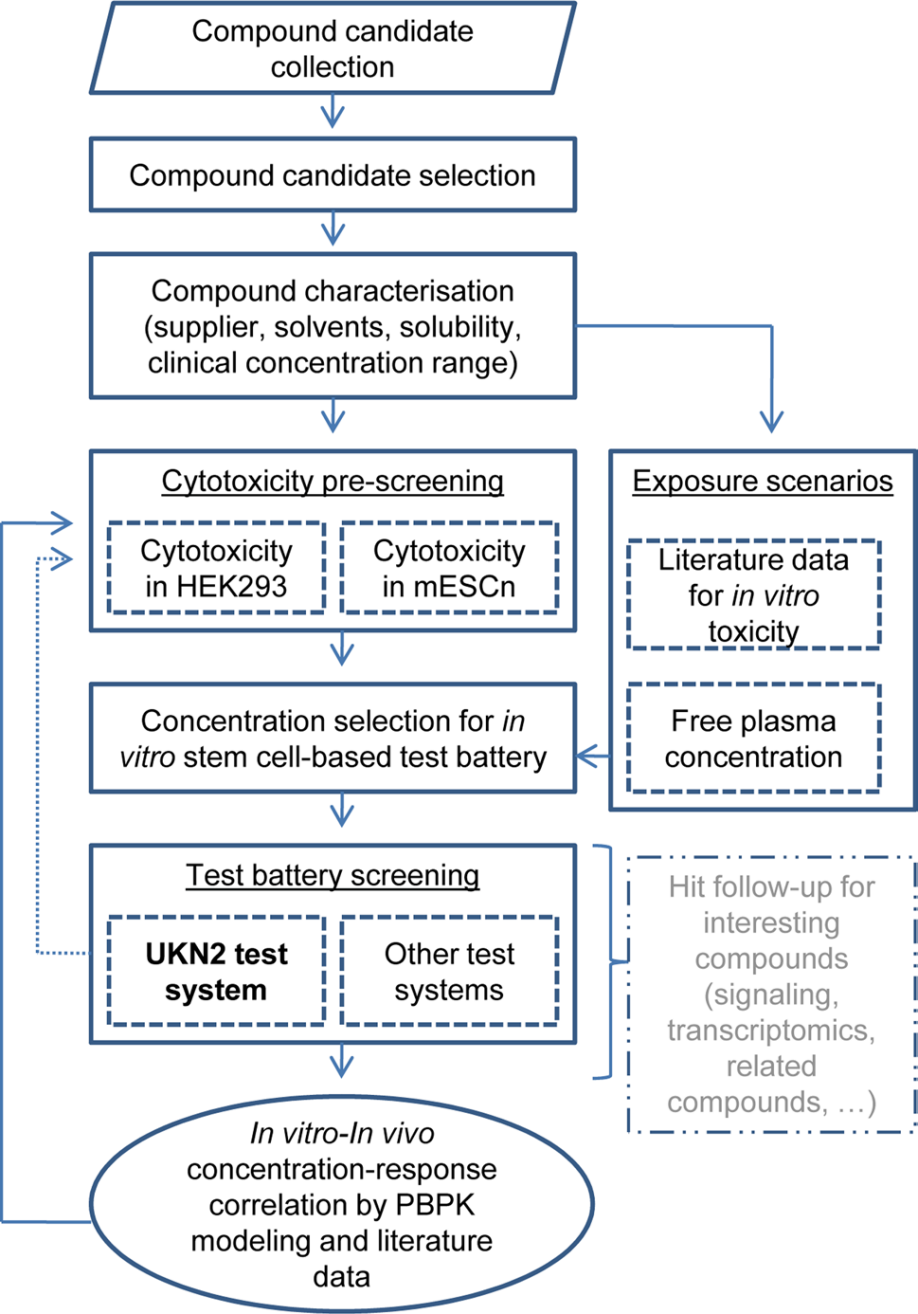
Overview of the ESNATS test battery. Candidate compounds were compiled based on criteria described in Fig. 2, and a final set of 28 drugs and environmental pollutants was selected. Their characteristics, including compound source, solubility, clinical concentration ranges and toxicological background information were compiled. Two approaches were chosen to determine a non-cytotoxic range for the further screening: cytotoxicity pre-screening on a transformed cell line derived from human embryonic kidney (HEK293) and on murine embryonic stem cell-derived differentiating neural cells (mESCn). In addition, realistic exposure concentrations were estimated from literature data mining (for in vitro toxicity information) and pharmacokinetic (PK) prediction (free plasma concentrations). The test battery comprised initially the UKN2 test system and three other hESC-based tests, but it was designed openly for any test addition. Screening proceeded in two steps: first the highest non-toxic or relevant concentration for a given test was determined; then compounds were tested in hESC models at this concentration. Based on the results of the screening, a shortlist of compounds was selected for further characterization by physiologically based pharmacokinetics (PBPK) modeling and for hit follow-up

**Fig. 2:**
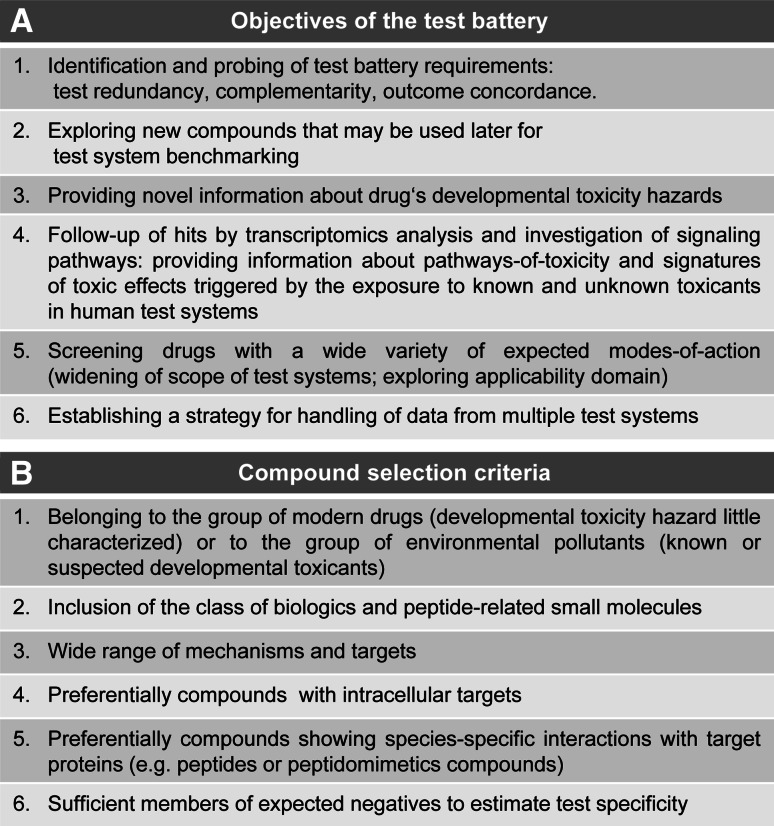
Test battery design criteria

Important features of the test battery framework are the characterization of the compounds concerning general cytotoxicity, relevant in vivo concentrations and other necessary background data. Accessory modules for hit follow-up and in vitro–in vivo extrapolation should provide rich information on many of the compounds in the future. In fact, one of the initial purposes of the test battery was the pre-filtering of hits for further toxicogenomics follow-up, for instance by transcriptome profiling (Figs. 1, 2a). This will be performed, once a sufficient number of hits will be characterized in different assays.

### Selection of test battery compounds

**Fig. 3:**
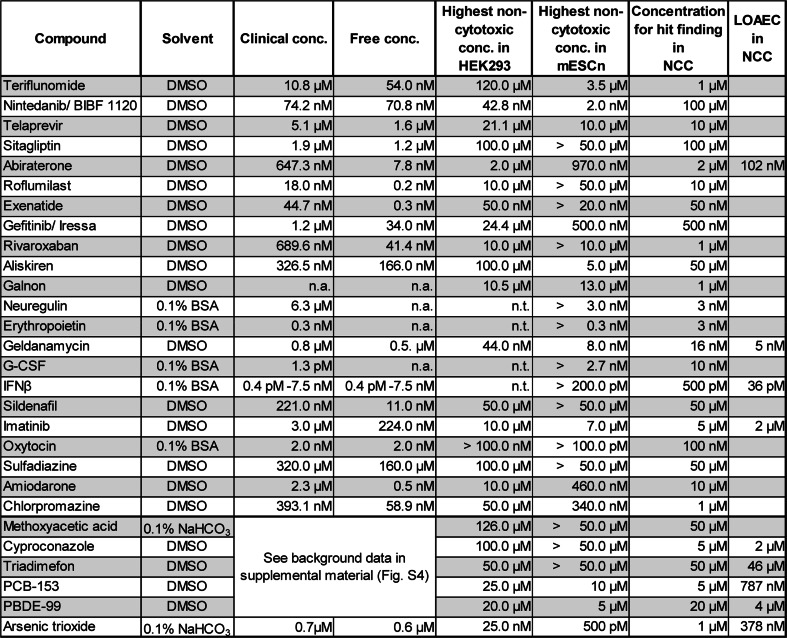
Toxicological background data for all compounds screened in the test battery. The solvent used for each compound is indicated. Data on clinical concentrations (maximal plasma concentration) and the free plasma concentrations are explained in greater detail in supplementary Fig. S4. The data obtained from the cytotoxicity pre-screening in HEK293 and mESCn cells are reported as the real data points closest to the mathematically modeled highest non-cytotoxic concentrations (BMCL15). The highest non-cytotoxic concentration determined in the UKN2 test system is also indicated. The *last column* indicates the lowest observed adverse effect concentration (LOAEC), the concentration triggering a 20 % inhibition of migration of the neural crest cells. (*n.a.* not available data, *n.t.* not tested)

The 28 compounds were compiled according to the selection criteria outlined in Fig. 2b. The test library reflects a compromise between the different criteria. Our choice marks a deliberate and intentional departure from the use of known toxicants and endpoint-specific controls (reviewed in Kadereit et al. 2012; Crofton et al. 2011; Leist et al. 2010), and it puts emphasis on the exploration of unknown drugs. Besides the drugs, a small selection (six substances) of environmental pollutants (e.g., PCB, PBDE, arsenic) was included as likely positive controls for many test systems. The group of drugs also included biologics (e.g., interferon-β, oxytocin) and peptide-related small molecules (e.g., sitagliptin, galnon). Some of the biologics were included as they are known to cross the blood brain barrier in vivo (e.g., G-CSF, erythropoietin). Finally, three compounds (sulfadiazine, chlorpromazine, amiodarone) were chosen because another drug screen (Kern et al. 2013) suggested a potential for developmental neurotoxicity. For all compounds of the test library, essential chemical and pharmacological information was compiled (Fig. S1, S2; Fig. 3). For environmental compounds with known neurotoxicity (developmental neurotoxicity) we referred to several pertinent in vivo and in vitro studies.

### Pre-screening of test battery compounds for general cytotoxicity

Most developmental neurotoxicity assays give reliable and specific results only when compounds are used at concentrations that do not trigger general cytotoxicity/cell death. This range has to be determined for each compound and each test system. However, most available assays allow only a relatively low throughput of samples. Therefore, it would be more efficient and economical to get some rough initial information on non-cytotoxic concentration ranges before the onset of testing. For this purpose, we used two different assays. The first was based on human HEK293 cells. Resazurin reduction was applied as viability endpoint after exposure to the test battery compounds for 48 h. The second assay made use of mESCn differentiated toward neural lineage. Also here, viability of the cells was determined by resazurin reduction after a 48 h exposure period during the initial stages of differentiation (starting on day 6). The combination of these assays was meant to cover many modes-of-action of cytotoxicants across species and cell biological functions.

**Fig. 4:**
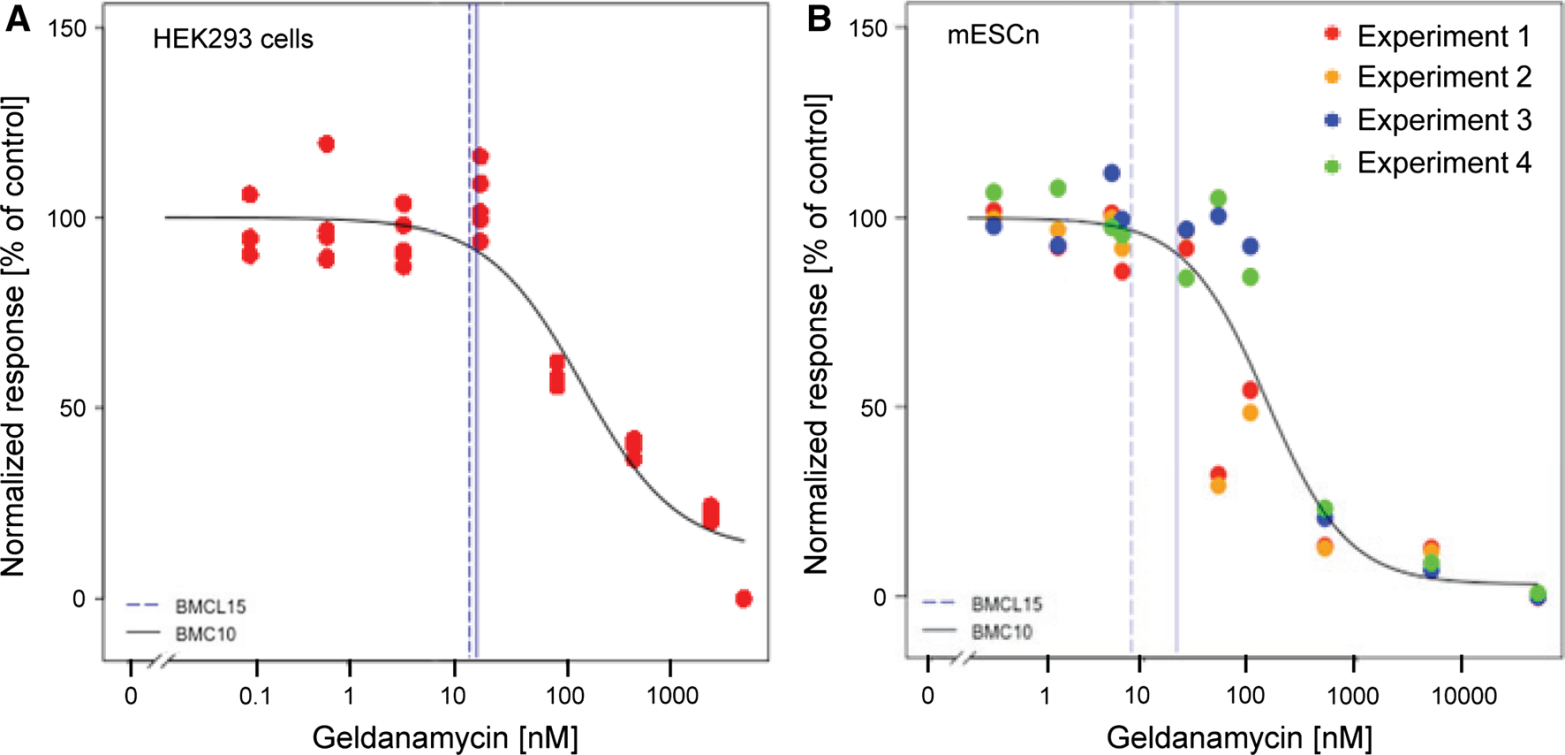
Determination of the highest non-cytotoxic concentration. Compounds were tested at multiple concentrations, and cell viability was determined by the resazurin assay (every replicate is represented by a *single circle*, different color code for independent experiments). These data were used to model a concentration–response curve and the concentration corresponding to a 10 % reduction in viability (BMC10) was calculated (*solid line*). In addition, the BMC15 was determined and the lower limit of its 95 % CI (BMCL15, *dashed line*) was determined. This latter value was used as estimate for the upper boundary of the non-cytotoxic concentration range. An example of BMC determination for the compound geldanamycin is shown, using data obtained from the cytotoxicity pre-screening in a HEK-293 cells and b mESC-derived differentiating neural cells (mESCn)

To determine the non-cytotoxic concentration range, compounds were tested at multiple concentrations. We then used a mathematical procedure to determine a benchmark concentration as upper limit of the non-cytotoxic range. The procedure is displayed in detail for the example compound geldanamycin (Fig. 4). For practical purposes, we used the real data point closest to this calculated theoretical threshold as toxicity threshold (Fig. 3). In most cases (16 compounds) in which comparative data were available, minimum cytotoxic concentrations of the compounds for the two cell types were similar (<5-fold difference). For all remaining substances (8 compounds), the embryonic stem cell system showed a higher sensitivity than the HEK293 cell line (Fig. 3). Cytokines were only tested at pharmacological concentrations to be expected in body fluids, and they all proved to be non-cytotoxic at these test concentrations (Fig. 3). In summary, the viability data give a rough indication on good starting points, but more precise data are needed for each new experimental model and for each experimental variation within a given test system (see below).

### Determination of toxicologically relevant concentration ranges

Besides non-cytotoxicity, further criteria are important to determine reasonable test concentrations and to interpret the data. To allow decisions on concentration ranges (e.g., for cytokines) and interpretation of screen results, we compiled the clinical blood and tissue concentrations (CC) for most compounds. These, together with data on plasma protein binding, were used to calculate the free plasma concentrations in patients. The latter data are important to relate in vivo data to in vitro concentrations. While an overview of the data is given in Fig. 3, more detailed information has been compiled in a supplementary table (Fig. S4).

For extrapolation of in vivo data to in vitro concentrations, often the issue arises, whether *C*_max_ (the peak concentration reached in clinics) or AUC (the average concentration found in a patient) should be used as anchor point for calculations. In the present study, both plasma peak (*C*_max_) and average data were considered. This allows case-by-case decisions, depending on the assumed mechanism of toxicity. For instance, it may be plausible that cytokine receptors need to be triggered only for a short time (by *C*_max_) to generate intracellular signals that potentially affect differentiation. In contrast to this situation, continuous cytokine signaling (in the AUC range) may be required to alter the cellular cytoskeleton and thereby to affect the cellular migration capacity. Vice versa, an environmental toxicant that inhibits a cellular pathway may need to do this continuously to exert developmental effects, i.e., an average toxic concentration (AUC) needs to be maintained to result in an average long-term inhibition. A short pulse, even at *C*_max_ may not be toxic. However, the situation would be different, if the compound (such as arsenite) binds irreversibly to cellular structures above a certain threshold concentration. Under such circumstances, the *C*_max_ values would become relevant.

### Inhibition of neural crest cell migration by test battery library

**Fig. 5:**
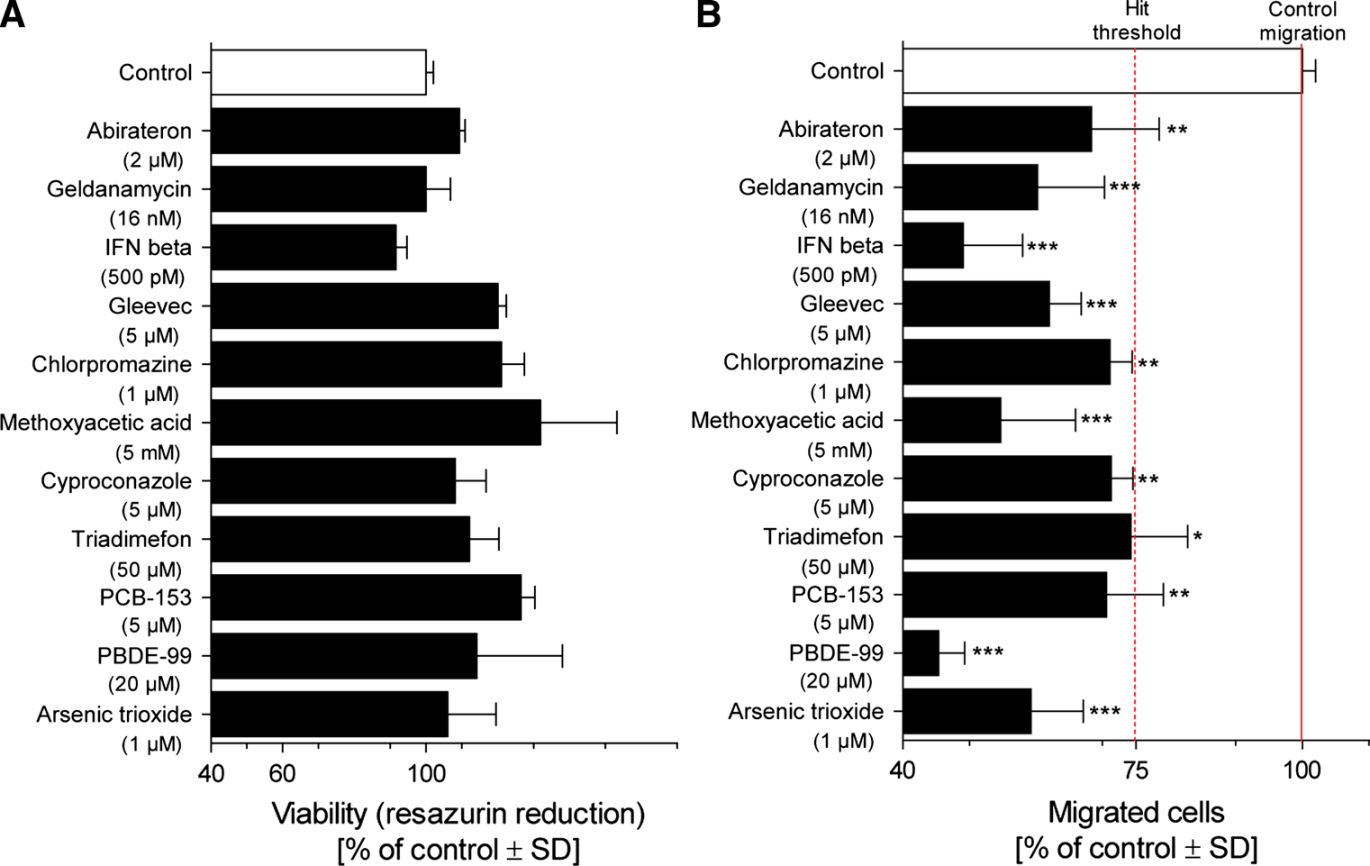
Overview of hits identified in the UKN2 test system. Neural crest cells were exposed to the highest non-cytotoxic concentration of test compounds for 48 h. The inhibition of cell migration and cytotoxicity induced by different compounds were measured by **a** the MINC and **b** resazurin assays. Substances leading to ≥25 % reduction in the NCC migration activity in the presence of ≤10 % cytotoxicity (compared with the untreated controls) were considered UKN2-positive hits. Data are mean ± SD of three independent experiments normalized to untreated controls. *p < 0.05, **p < 0.01, ***p < 0.001

**Fig. 6:**
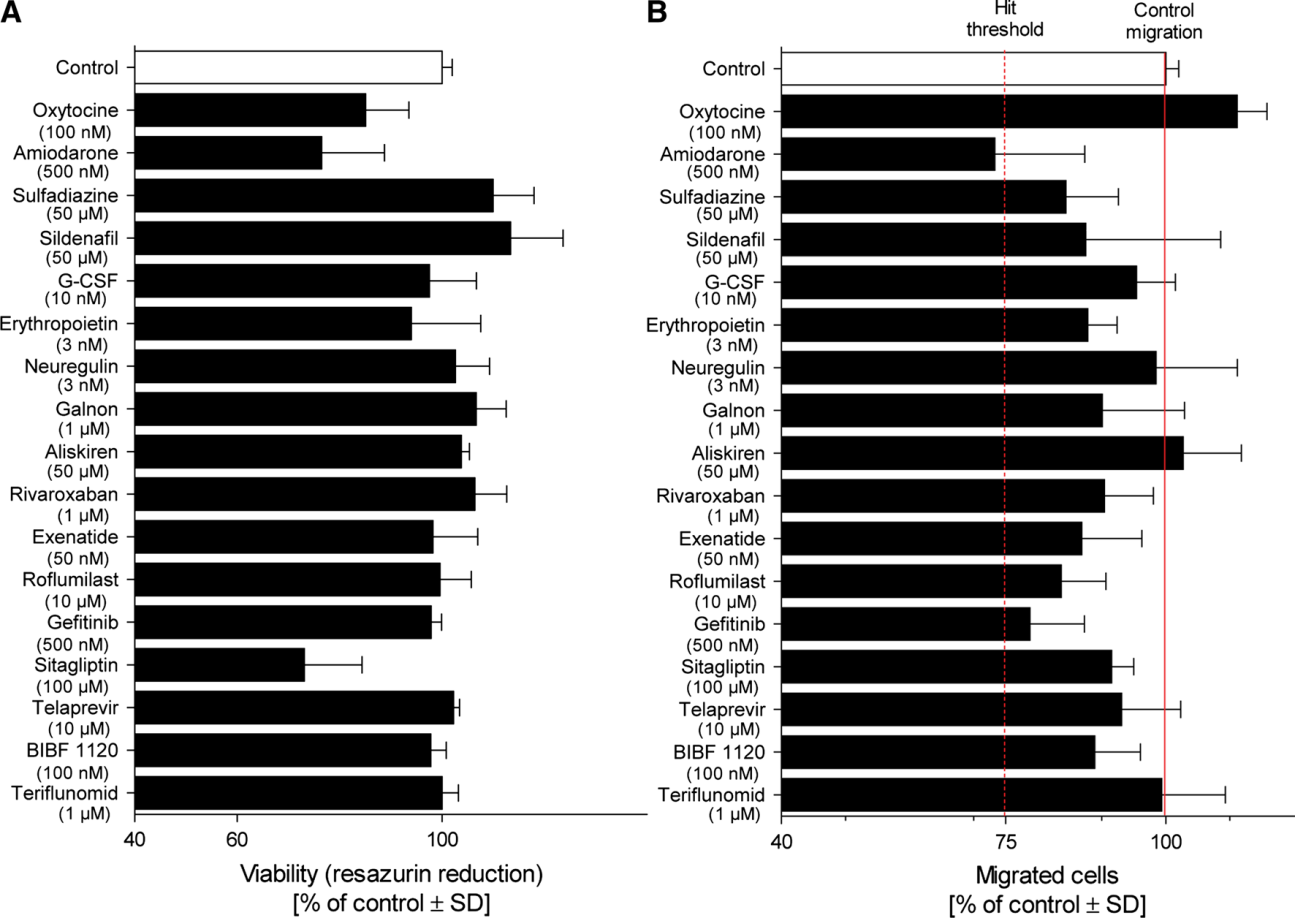
Overview of negative compounds in the UKN2 test system. The results of a 48 h exposure of neural crest cells to the highest non-cytotoxic concentration of test compounds are shown. The inhibition of cell migration and cytotoxicity induced by different compounds were measured by **a** the MINC and **b** resazurin assays. Substances showing reduction in cell viability (>10 %) and/or reduction in migration <25 % (compared with the control) were considered UKN2-negative compounds. Data are mean ± SD of three independent experiments normalized to untreated controls. *p < 0.05, **p < 0.01, ***p < 0.001

As first assay to be run within the framework of the test battery, we chose the UKN2 test system that evaluates interference of potential toxicants with neural crest (NC) cell migration (Zimmer et al. 2012). For the primary screen, the library was run at a single concentration, selected according to the cytotoxicity pre-screening. At this concentration the viability of NCC was determined. If the concentration was non-toxic for NCC, then inhibition of migration was determined in the MINC assay. If the compounds were initially toxic, the UKN2 toxicity threshold was determined and then the MINC assay was performed at this concentration. When comparing cytotoxicity tests across different cell lines and models, we observed that cytotoxicity depends not only on the cell type and the medium used. Often minor experimental details affected the outcome (e.g., the plate type used). Therefore, the most relevant cell viability data were obtained when this endpoint was measured in each test run, and directly within the sample used for measuring the specific endpoint (e.g., migration). For instance, about two-thirds of the test compounds showed similar cytotoxicity thresholds in the pretesting models (HEK293; mESCn) and the MINC. For two compounds, cytotoxicity of the NCC resembled rather the mESC than HEK293; for three other compounds, NCC sensitivity was closer to that of HEK293. After identification of adequate concentrations viability and MINC data were obtained for each compound, and experiments were repeated at this test concentration with two further cell preparations (Figs. 5, 6). Hit compounds (Fig. 5) reduced the migration for at least 25 %, while they reduced cell viability in the same assay for <10 %. Non-hits did either not affect migration strongly, or they influenced migration only above the cytotoxicity threshold (Fig. 6). Altogether 11 hits were identified, 5 of which came from the group of drugs. Within the latter group, the hit rate was 23 %; among the environmental compounds, the hit rate was 100 %.

### Determination of the minimal concentration triggering developmental toxicity

**Fig. 7:**
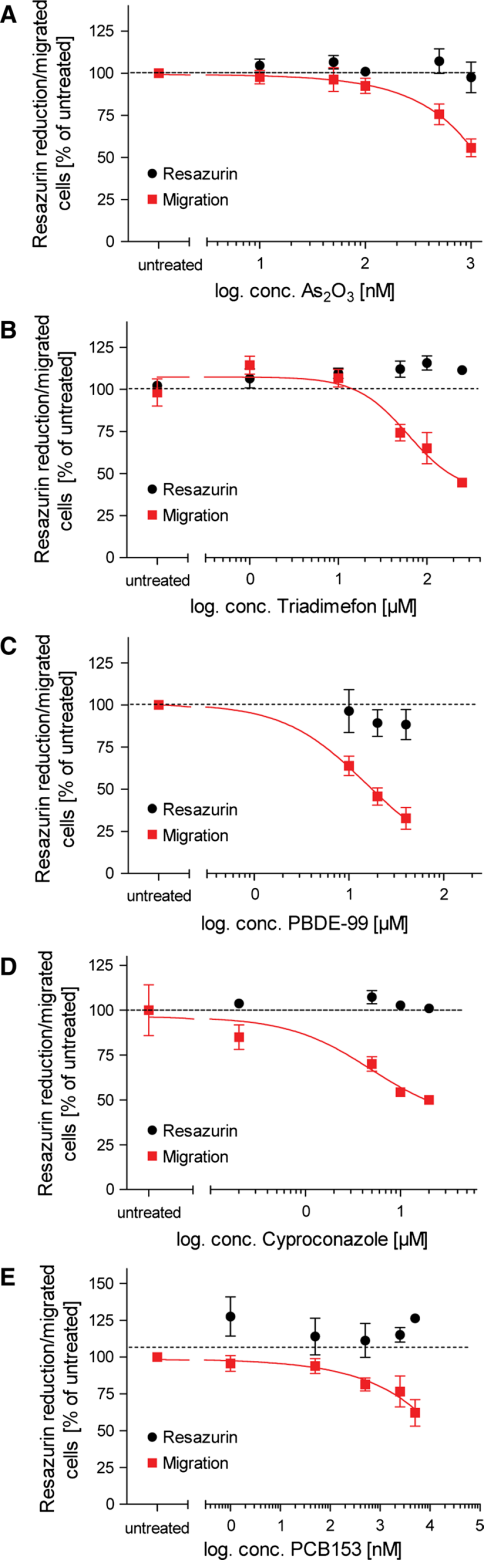
Concentration-response curves for UKN2-positive environmental pollutants. Neural crest cells were exposed to different concentrations of each test compound for a period of 48 h. The inhibition of cell migration (*red squares*, *solid line*) and cytotoxicity (*black dots*) induced by different compounds were measured by the MINC and resazurin assays. **a** As_2_O_3_, **b** triadimefon, **c** PBDE-99, **d** cyproconazole and **e** PCB153 showed a concentration-dependent effect on the NCC migration, in a not-cytotoxic concentration range. Data are mean ± SD of three independent experiments normalized to untreated controls. *p < 0.05, **p < 0.01, ***p < 0.001 (color figure online)

**Fig. 8:**
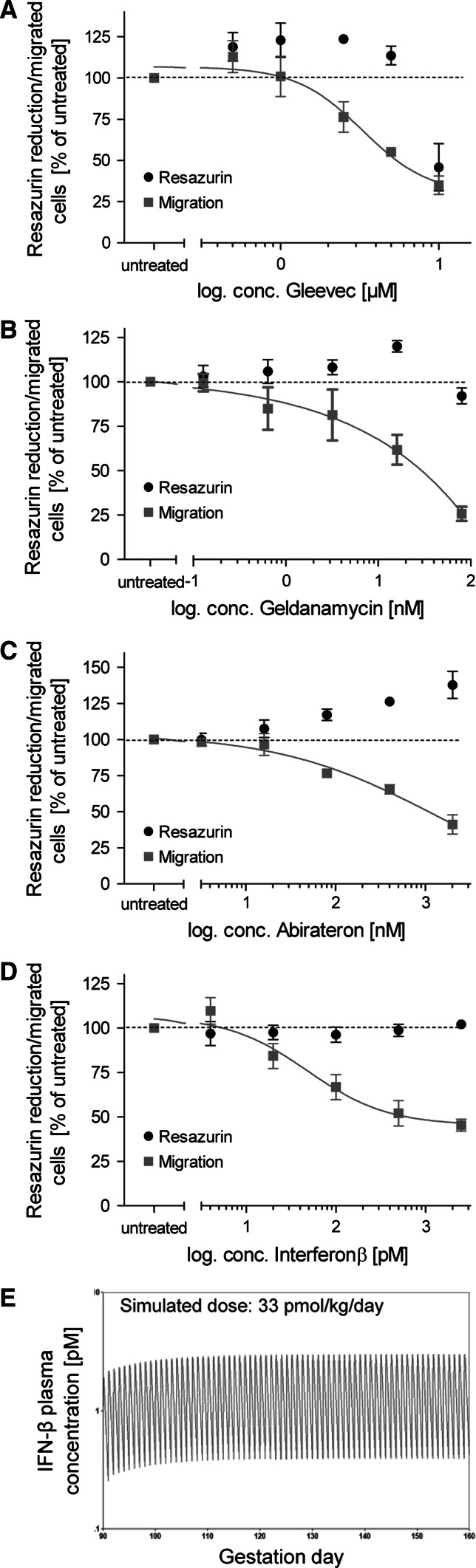
Concentration-response curves for drugs identified as hits in the UKN2 test system. Neural crest cells were exposed to various concentrations of test compounds for a period of 48 h. The inhibition of cell migration (*red squares*, *solid line*) and cytotoxicity (*black dots*) induced by different compounds were measured by the MINC and resazurin assays. **a** Gleevec, **b** geldanamycin, **c** abiraterone and **d** interferon β (IFN-β) showed a concentration-dependent effect on the NCC migration, in a non-cytotoxic concentration range. Data are mean ± SD of three independent experiments normalized to untreated controls. (*p < 0.05, **p < 0.01, ***p < 0.001). **e** Simulation of IFN-β plasma concentration induced by a sub-cutaneous dose of 33 pmol/kg/day from GD90 (gestation day 90) to term (GD160) in cynomolgus monkeys, using a PBPK model published by Mager et al. (2003) and reconstructed in acslX software (color figure online)

To follow up on the hits from the initial screen by the MINC assay, a broader range of concentrations of these compounds was tested in the same assay. We used these data to determine the lowest concentration inhibiting the specific test endpoint—neural crest cell migration. A nonlinear regression was fitted to the concentration–effect curve (Figs. 7, 8). For this type of follow-up assay, we assumed that a reduction in migration by 20 % was of biological and toxicological significance. The concentration triggering this extent of inhibition was determined and defined here as the ‘lowest observed adverse effect concentration’ (LOAEC). This value represents the lowest concentration at which inhibition of migration would become toxicologically relevant in our test system (Fig. 3).

### Examples of PBPK modeling for hit compounds to relate in vitro correlations to realistic exposure scenarios

We selected three compounds for more detailed comparisons of LOAEC values and toxicologically relevant in vivo concentrations. First, recombinant human IFN-β was examined. A PBPK model for the analysis of IFN-β kinetics in monkeys has been described by Mager et al. (2003). In this study, IFN-β plasma concentrations were measured during a period of 48 h in order to determine the pharmacokinetic and pharmacodynamic parameters necessary for modeling. The model of Mager et al. (2003) postulates a decrease in receptor density upon repeated exposure to IFN-β, and an elimination of IFN-β (internalized into cells) depending on the receptor density. We implemented this PK model in the acslX software, and we simulated the kinetics of IFN-β after exposure to a dose known to trigger developmental toxicity in animals: this simulated toxic dose was chosen based on an unpublished report summarized by the US FDA (see Materials and Methods). In this report, developmental toxicity in pregnant monkeys exposed to IFN-β was shown for a dose of 740 ng/kg/day (= 33 pmol/kg/day, assuming a molecular weight of 22.5 kDa) given from gestational day 90 to term (GD160). Our simulations showed for a subcutaneous dose of 33 pmol IFN-β/kg/day that receptor density decreased by about one-third. As a consequence, daily average plasma concentrations of IFN-β increased from 0.9 to 1.5 pM. The simulated plasma peak concentrations were in the range of 2–3 pM (Fig. 8e). This value was about tenfold lower than our in vitro NOAEL (no observed adverse effects level), i.e., still within the same order of magnitude.

**Fig. 9:**
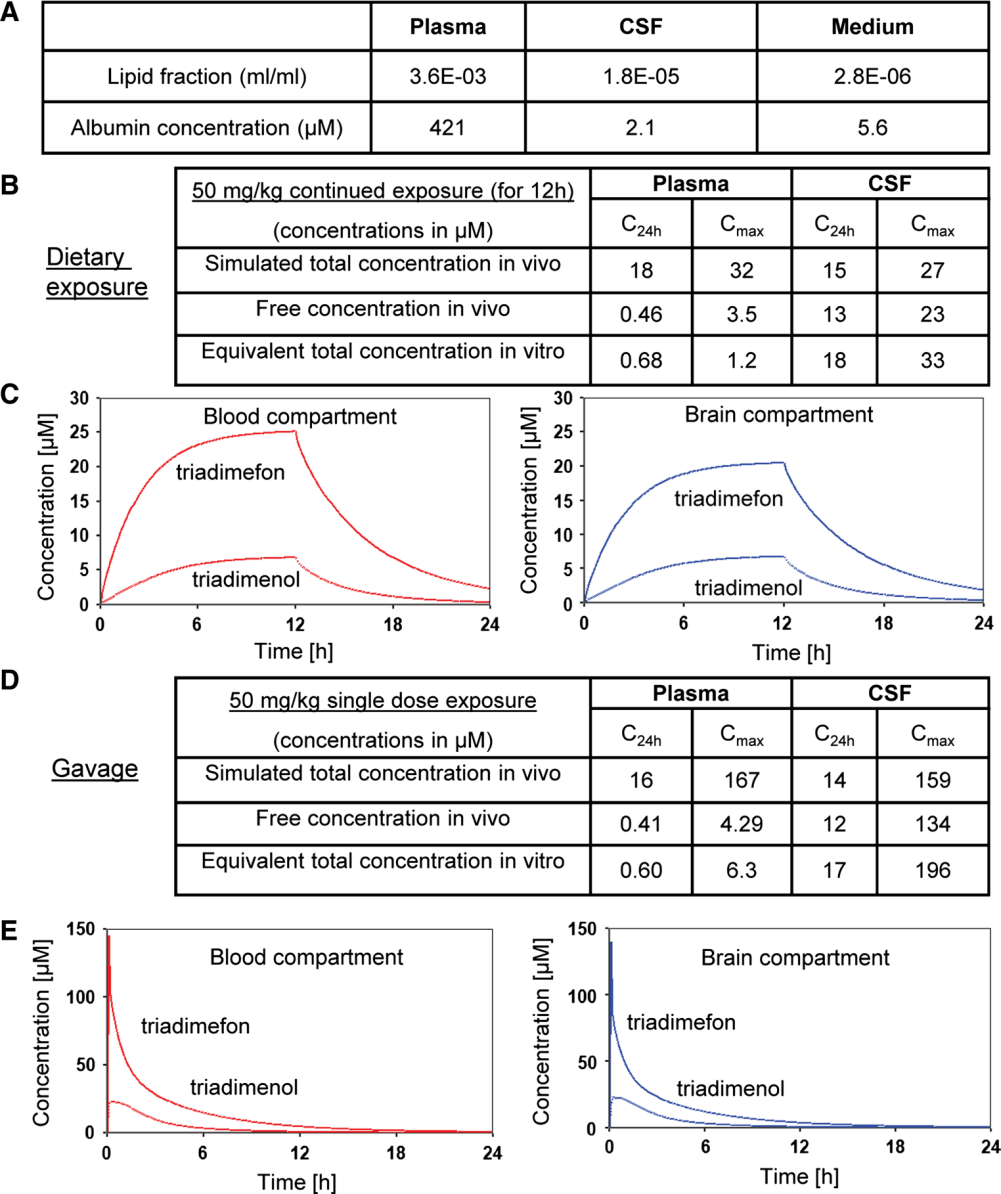
PBPK modeling of the pesticide triadimefon **a** The values of lipid fractions and albumin concentrations in rat plasma, cerebrospinal fluid (CSF) and in vitro cell culture medium used to calculate the equivalent in vitro concentrations are listed. **b**–**e** The toxicokinetic behavior of triadimefon and its metabolite triadimenol was simulated by using a PBPK model published by Crowell et al. (2011). Two different exposure scenarios are shown: **b**, **c** a dietary exposure of 50 mg/kg triadimefon (intake for the first 12 h within a 24 h period) and **d**, **e** a single dose of 50 mg/kg triadimefon administered by gavage. **b** The maximum concentrations (*C*_max_) and the average concentrations (*C*_24h_) calculated for plasma and CSF are indicated for the first scenario: these values together with data in **a** were used to calculate the free concentration in vivo and the equivalent total concentration in vitro. **c** The simulated concentration of triadimefon and triadimenol in rat blood and brain compartment over time is shown for an oral dose of 50 mg/kg taken in the diet during the first 12 h of a 24 h period. **d** The maximum concentrations (*C*_max_) and the average concentrations (*C*_24h_) calculated for plasma and CSF are indicated for the second scenario: these values together with data in **a** were used to calculate the free concentration in vivo and the equivalent total concentration in vitro. **e** The simulated concentration of triadimefon and triadimenol in rat blood and brain compartment over time is shown for a single dose of 50 mg/kg

Triadimefon was the second compound selected for PK modeling because it represents a specific NCC toxicant (Zimmer et al. 2012; Menegola et al. 2005). Further knowledge on the mechanisms of toxicity would be of high interest. We revisited/reconstructed the PBPK model by Crowell et al. (2011) to predict the target tissue concentrations related to the exposure scenarios leading to published toxic effects on male fertility and the CNS (Goetz et al. 2007; Crofton et al. 2011), i.e., a dose of 50 mg/kg triadimefon. The maximum simulated total in vivo concentration (*C*_max_) and its average in the 24-h period (*C*_24h_) in plasma and brain were used as relevant exposure metrics. Equal contribution of triadimefon and its metabolite triadimenol to the observed effects relative to their concentrations was assumed; therefore, the concentrations of both compounds were added. The obtained in vivo total concentration values were used in order to assess the equivalent in vitro concentrations (Fig. 9a). We simulated two scenarios: dietary exposure assuming a constant intake of the entire drug dose within the first 12 h of a 24-h period (Fig. 9b,c); oral gavage, modeled as a bolus dose into the liver compartment (Fig. 9d,e). The averaged *C*_24h_ simulated in vivo in plasma after dietary exposure was estimated to be 18 μM, with a *C*_max_ of 32 μM. *C*_24h_ and *C*_max_ in CSF were accordingly 15 and 27 μM. Based on the parameters listed in Fig. 9a and on the equations reported in Materials and Methods, we calculated the free concentration in vivo and the equivalent total concentration in vitro (about 1 μM in plasma, and 18–33 μM in CSF) (Fig. 9b). The same approach was used for the second analyzed scenario. A single dose of 50 mg/kg of triadimefon administrated by gavage was simulated in our model. The equivalent in vitro concentrations were 17–196 μM (Fig. 9d). This illustrates that in vivo plasma concentrations were within the range of the MINC NOAEL.

The third compound studied was PBDE-99. A new PBPK model was established for this purpose (Fig. S3). It was used to simulate concentrations in the rapid equilibrium compartment (e.g., plasma) corresponding to the lowest exposures related to neurodevelopmental toxicity described in Kuriyama et al. (2005) and Viberg et al. (2005). An oral dose of 0.11 μmol/kg (on gestational day 6, as used in the first study) was simulated to lead to a maximum concentration of 0.1 μM in our model (Fig. S5a). An oral dose of 1.4 μmol/kg (on postnatal day 10, as used by Viberg et al. 2005) led to a simulated maximum concentration of 1.3 μM, with an average concentration of 0.82 μM over the first 10 days after exposure (Fig. S5a). These concentrations were within the same order of magnitude as the MINC NOAEL.

## Discussion

In the present study, we defined the framework for an in vitro developmental toxicity test battery. Although it was originally designed for assay systems that have been developed by the ESNATS consortium (Leist et al. 2013), it may be expanded by any other robust test. Additionally, we performed a feasibility study. For this, we chose the MINC assay (Zimmer et al. 2012), because its throughput and its functional endpoint allowed us to judge the suitability of the test strategy and compound library within a reasonable time frame. The environmental chemicals with known DNT hazard potential that were included into the screen were all identified as hits. This confirmed the very high sensitivity of the assay. More importantly, new hits were identified among the group of medical drugs. This points to the usefulness of the test battery to provide safety information on hitherto non-characterized drugs or environmental compounds.

The field of developmental neurotoxicity relies on only a handful of generally accepted positive controls, such as mercury, lead and pesticides (Grandjean and Landrigan 2006; Kuegler et al. 2010; Kadereit et al. 2012). This situation is due to a lack of comprehensive human epidemiological data as well as a dearth of DNT guideline studies on animals (Rovida et al. 2011; Makris et al. 2009; van Thriel et al. 2012). Therefore, instead of solely relying on the compounds that have been used in the past, we applied a new approach by combining well-known positive controls with new compounds mainly belonging to the group of medical drugs. The test battery approach gives the opportunity to qualify this new group of compounds by non-tiered testing in multiple assays covering different key biological processes. We envisage that this approach will yield some extremely well-characterized drug compounds, which could then be used in the future to increase the list of positive controls for in vitro assays. Moreover, performance of the assays would be characterized by their hit patterns and by mechanistic studies on the hits in the respective test systems.

Although we acknowledge that our approach has weaknesses concerning the definition of positive controls, it provides nevertheless a new opportunity for breaking a vicious circle between lack of sufficient reference compounds and a paucity of validated assays. We feel that this strategy is worth being explored considering also that this approach has been successful in other fields. For instance, the area of chemically induced carcinogenesis faces similar problems, despite more than 1,000 times larger research efforts. The list of IARC (International Agency for Research on Cancer) group 1 compounds (definite human carcinogens) contains only about four dozen chemicals. Considering the heterogeneity of cancer, this number is far too low to validate any assay by conventional correlative statistics. Nevertheless, many assays have been developed, and many chemicals have been classified as potential carcinogens. This was done by recurrent optimization cycles involving testing of compounds, assay optimization, and adaptation of interpretation models. Human data are hard to obtain for chemically induced carcinogenesis, but some key biological processes responsible for human carcinogenesis, such as mutagenesis, promotion of growth, loss of contact inhibition can be defined and tested individually in vitro (Adler et al. 2011). This procedure and experience may be transferred to the field of DNT. We therefore believe that increasing the size and heterogeneity of the test compound library as well as comparing the toxicity data obtained in several assays for key biological processes will be a successful strategy to overcome the lack of positive controls for DNT/DT.

By adding the new group of medical drugs to the test battery library, we also addressed a further issue: applicability domains of most existing assays are poorly defined. The currently available test systems have been characterized by the use of well-known positive control compounds, which mainly belong to the group of environmental toxicants (Klaric et al. 2013; Vojnits et al. 2012; Krug et al. 2013a; Pallocca et al. 2013; Laurenza et al. 2013; Piersma et al. 2013; Bal-Price et al. 2012; Crofton et al. 2011; Coecke et al. 2007; Lein et al. 2007; Kadereit et al. 2012). Some others have used at most one to three drug-like compounds (Balmer et al. 2012; Stiegler et al. 2011; Kuegler et al. 2012; Meganathan et al. 2012; Jagtap et al. 2011; Falsig et al. 2004). Therefore, it is not clear whether these test systems are able to predict DT of drugs or specific groups of environmental compounds. Apart from two screening assays developed in the context of ESNATS (Kern et al. 2013; Krug et al. 2013a) the published assays have never been challenged by a broad range of drugs, and they may therefore not have been optimized to detect their adverse effects. However, the pharmaceutical industry would need such new assays capable to predict human DT in a more reliable fashion than the currently available tests. In the past years, pharmaceutical companies have struggled more and more to develop new drugs, while at the same time drugs already on the market had to be withdrawn due to safety issues. Between 1999 and 2011, 19 % of 279 newly approved drugs in Europe were reported to have post-approval safety issues and five drugs had to be withdrawn from the market (Mol et al. 2013).

The strategy used here also attempts to provide a new perspective on how the field of in vitro DT testing may advance with regards to future validation of assay systems. The classical situation foresees the comparison of in vitro toxic concentrations with the known in vivo toxic doses, as major indication of biological relevance. This approach is not suitable for developmental toxicity testing since not enough animal data are available. As alternative, a mechanistic validation has been proposed (Leist et al. 2008b, 2012b; Hartung et al. 2013). This concept is based on the assumption that toxicants disrupt key biological processes, and that test systems identify compounds that disrupt such processes (Leist et al. 2010; Crofton et al. 2011; Kadereit et al. 2012). For instance, cell migration represents such a key process (Fritsche et al. 2011; Moors et al. 2009), and inhibition of precursor cell migration in the nervous system may lead to persistent and externally visible DT. If a DNT assay is designed to identify such effects, its validity estimate would increase, as soon as it can be shown that the test is based on cell biological and signaling processes controlling precursor cell migration. Validity would increase even more, if it can be demonstrated that compounds interfering with such processes are identified as hits in the test system. Furthermore, it would be important for mechanistic validation to demonstrate that chemicals score as hits because they interrupt such defined processes, and that toxicity may be rescued by a defined, mechanistically understood counter-regulation (Krug et al. 2013a; Zimmer et al. 2011b; Poltl et al. 2012; Wang et al. 2007; Volbracht et al. 1999; Wayman et al. 2012a, b; Schildknecht et al. 2013). The evaluation of such a test system would thereby be independent of classical positive controls; it would solely be based on knowledge regarding toxicity pathways. Our test battery framework would allow such approaches under the following conditions: first, the same compounds will be tested in different, mechanistically defined test systems; by evaluating different endpoints and comparing the data obtained from different assays, more information will be gained on the general mode of action of those toxicants. The testing strategy needs to reach beyond the primary screening of toxicants to document adverse effects; additional mechanistic characterization in secondary assays and whole-genome transcriptomics analysis of hits is an essential part of the strategy. This should promote our understanding of the pathways mainly affected by the exposure to the toxicants and of the pathways responsible for the toxicity.

Apart from the compound selection, we took a second unconventional approach with respect to the choice of compound test concentrations. In drug discovery, it has been general practice to screen compounds at fixed absolute concentrations. In contrast to this, we used relative concentrations. Biological activity of the compounds in vitro and in vivo, e.g., cytotoxicity, and clinical plasma concentrations were included as reference. Testing was performed relative to such reference concentrations. In most cases, the non-cytotoxic range was evaluated and chosen as starting point for the screen. However, depending on the compounds, other criteria have been considered as more appropriate; this has been the case for cytokines that were tested in concentration ranges corresponding to the levels expected in body fluids during clinical application. This strategy was designed for test systems whose throughput is limited to some extent. For assays with very high throughput, an alternative approach would be to simply screen a large number of concentrations over the entire range of compound solubility. This approach is taken for instance by the national toxicology program of the USA (Xia et al. 2008; Attene-Ramos et al. 2013; Tice et al. 2013) or the EPA ToxCast program (Judson et al. 2010; Sipes et al. 2013).

The current data are only derived from a first screening step. In the future one could imagine linking the functional disturbance of substances found in the MINC with more mechanistic data obtained in the assay itself, but also in other tests within the framework. This would allow to deepen our understanding of possible pathways of toxicity. Using the MINC assay in the first initial screen allowed us to identify 23 % of compounds belonging to the drug group as potential DNT/DT toxicants. On the other hand, more than 70 % of the drugs in our library did not show any specific effect, although they have potent biological activity in many other tests. This suggests that the MINC assay does not react entirely unspecifically to any drug-like compound or biologic.

Among the newly identified hits, we found three anticancer drugs (abiraterone, geldanamycin and imatinib), an anti-psychotic drug (chlorpromazine) and a cytokine, mostly used for multiple sclerosis treatment (IFN-β). Abiraterone and imatinib have been shown to promote remission of different metastatic cancers (Patel 2013). Possibly, such findings are related to an inhibition of migration of cancer cells as well, and this may be tested in the future. Also in the case of the HSP90 inhibitor geldanamycin, other studies agree with our findings in the MINC assay. The effect of the drug on cell migration has been studied in several cancer cell lines. Activity of focal adhesion kinases, actin reorganization and integrin activation was inhibited by geldanamycin in bladder carcinoma cells (Koga et al. 2007). Furthermore, chemotactic activity in sarcoma cells (Lesko et al. 2007) as well as migration of glioma cells was reduced (Zagzag et al. 2003). Also the dopamine antagonist chlorpromazine, has been shown by other studies to inhibit migration in a pancreatic carcinoma cell line, via inhibition of k-RAS (Eisenberg et al. 2008).

Interference of IFN-β with migration has also been shown in several in vitro studies; the cytokine seems to modulate the activity of chemokine receptors and matrix remodeling proteases (e.g., CCR7, MMP9) in dendritic and immune cells (Yen et al. 2010; Stuve et al. 1996). So far, these mechanistic findings have not been corroborated in an in vivo setting, but the drug showed potential DNT activity in in vivo studies (US FDA, see Materials and Methods). Modeling the pharmacokinetics of the drug, we found that the toxic effect is triggered by a blood concentration of 1.9–3 pM. About 100-fold higher concentrations may be reached upon bolus injections (i.v.) (Yung et al. 1991), and the LOAEC observed in the MINC assay is about in the middle of this concentration range. The molecular targets responsible for the adverse effect of IFN-β seen in those and in our study are not known. We hope that further characterization of the compound in the MINC assay and other assays of the battery will provide more details regarding the mode of action relevant for toxicity.

For the future, we suggest that the compound library may be expanded, and data from different publications and laboratories may be stored in a common database. It cannot be stressed enough that initial hit-finding is only the first step of this strategy. Complementary information from multiple assays, pharmacokinetic modeling and mechanistic follow-up studies are necessary components of the overall framework. For instance, transcriptome data and measurement of other Omics and functional endpoints (Bouhifd et al. 2013; Hogberg et al. 2011; Ramirez et al. 2013; Lefew et al. 2013) will expand our knowledge regarding the toxicity mechanisms of chemicals. Moreover, such data will serve as a knowledge base concerning the biological processes and signaling pathways that need to be covered by a future DNT test battery. This will help us to identify redundancies, complementarities, strengths, weaknesses, applicability domains and limitations of current individual assays with regards to their predictive value for DNT.

## Electronic supplementary material

Below is the link to the electronic supplementary material.

Supplementary material 1 (PDF 447 kb)

## Abbreviations

ADME: Absorption, distribution, metabolism, excretion
bFGF: Basic fibroblast growth factor
BMC: Benchmark concentration
BMCL: Benchmark concentration 95 % confidential interval lower limit
CC: Clinical concentration
CCR: Chemokine receptors
CNS: Central nervous system
CSF: Cerebrospinal fluid
CYP: Cytochrome P450
Dll: Distal less
DNT: Developmental neurotoxicity
DT: Developmental toxicity
EGF: Epidermal growth factor
ESNATS: Embryonic stem cell-based novel alternative tests
FCC: Free concentration
GD: Gestational day
HEK293: Human embryonic kidney 293 cell
hESC: Human embryonic stem cell
IFN-β: Interferon β
LOAEC: Lowest observed adverse effect concentration
MEF: Mouse embryonic fibroblast cell
mESCn: Murine embryonic stem cell-derived neural precursor
MINC: Migration of neural crest cell
MPP: Matrix metalloproteinases
NCC: Neural crest cell
NOAEL: No observed adverse effect level
PBDE: Polybrominated diphenyl ether
PBPK: Physiology-based pharmacokinetic
PCB: Polychlorinated biphenyl
ROI: Region of interest
