# Supplementary material for: Profiling of drugs and environmental chemicals for functional impairment of neural crest migration in a novel stem cell-based test battery
Source: Arch Toxicol. 2014 Apr 2;88(5):1109–26. doi: 10.1007/s00204-014-1231-9 (PMC3996367; doi:10.1007/s00204-014-1231-9)
Supplement: Supplementary file 1 — Supplementary material 1 (PDF 447 kb) [file 204_2014_1231_MOESM1_ESM.pdf]

Supplemental Material

Profiling of drugs and environmental chemicals for functional impairment of neural crest migration in a novel stem cell-based test battery

B Zimmer, G Pallocca, N Dreser, S Foerster,T. Waldmann, J. Westerhout, S Julien, KH Krause, C van Thriel, J. Hengstler, A.Sachinidis, S Bosgra, M Leist

Table of contents

- **Supplemental Material, Figure S1** **page 2**
  Chemical and pharmacological characteristics of the group of medical drugs

- **Supplemental Material, Figure S2** **page 5**
  Chemical and pharmacological characteristics of the group of environmental pollutants

- **Supplemental Material, Figure S3** **page 6**
  PBPK modeling for PBDE-99

- **Supplemental Material, Figure S4** **page 7**
  Information about clinical concentration ranges and free concentration ranges for chemicals of the test battery compound list

- **Supplemental Material, Figure S5** **page 16**
  Simulation of PBDE-99 toxic exposure by PBPK modeling

- **Supplemental Bibliography** **page 17**

Supplemental Material, Figure S1:
Chemical and pharmacological characteristics of the group of medical drugs

Compound	Pharmacological characteristics	Chemical characteristics	Supplier/ Catalog No.
Teriflunomide 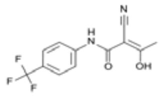	Immunomodulatory drug, inhibiting pyrimidine de novo synthesis by blocking the enzyme dihydroorotate dehydrogenase	Amide	Enzo Life sciences/ ALX-430-096-M005
Nintedanib 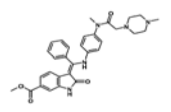 (BIBF1120;Vergatef)	Tyrosin kinase inhibitor developed for tumor therapy, inhibits signaling of three growth factor receptors involved in angiogenesis (VEGFR, PDGFR and FGFR)	Nucleotide mimetic	Selleckchem/ S1010
Telaprevir 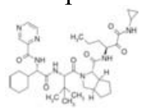	Antiviral drug for the treatment of hepatitis C; protease inhibitors	Peptide mimetic	Selleckchem/ S1538
Sitagliptin 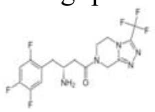	Oral anti-hyperglycemic (antidiabetic) of the dipeptidyl peptidase-4 (DPP-4) inhibitor class	Peptide mimetic	Selleckchem/ S4002
Abiraterone 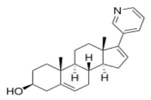	Antiandrogen; inhibits 17 α-hydroxylase/C17,20 lyase (CYP17A1), an enzyme which is involved in steroid (testosterone) synthesis	Steroid	Selleckchem/ S1123
Roflumilast 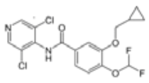	Selective, long-acting inhibitor of PDE-4; isoform of phosphodiesterases	Benzamide	Selleckchem/ S2131
Exenatide 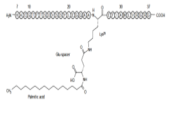	Glucagon- like peptide-1 receptor agonist (GLP-1 mimetic); treatment of diabetes mellitus type 2	Peptide/Protein	Prospec/ HOR-246
Gefitinib 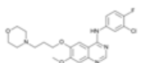 (Iressa)	Inhibitor of epidermal growth factor receptor's (EGFR) tyrosine kinase domain	Nucleotide mimetic	Selleckchem/ S1025

Rivaroxaban 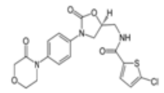	Oral anticoagulant; inhibition of the factor Xa protease	Peptide mimetic	Selleckchem/ S3002
Aliskiren 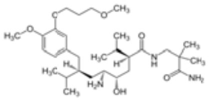	Inhibitor of renin protease; treatment of essential (primary) hypertension (preventing of the conversion of angiotensinogen to angiotensin I)	Peptide mimetic	Selleckchem/ S2199
Galnon 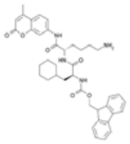	Selective agonist at the galanin receptors GALR. Anticonvul sant, anxiolytic, anorectic and amnestic effects in animal models	Peptide mimetic	Sigma-Aldrich/ G4419
Neuregulin 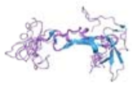	Endogenous agonist of the erbB family of tyrosine kinase receptors; plays multiple essential roles in neuronal development and disease	Peptide/Protein	R&D Systems/ 378-SM-025
Erythropoietin 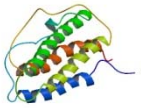	Glycoprotein hormone that acts as agonist of EpoR and controls erythropoiesis and neurogenesis	Peptide/Protein	R&D Systems/ 287-TC-500
Geldanamycin 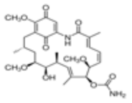	Benzoquinone ansamycin antibiotic that binds to Hsp90 (heat shock protein 90) and inhibits its function. Antitumor effects by acting on v-Src, mutant p53 proteins, Raf-1 and EGFR signalling	Amide	Selleckchem/ S2713
G-CSF 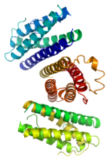	Protein hormone that stimulates granulopoiesis and with neuroprotective effects	Peptide/Protein	R&D Systems/ 214-CS-005
IFNβ	Protein hormone (cytokine); multiple sclerosis treatment	Peptide/Protein	R&D Systems/ 11415-1
Sildenafil 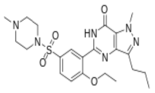	Inhibitor of the PDE5 isoform of phospodiesterases; used to treat erectile dysfunction and pulmonary arterial hypertension (PAH).	Nucleotide mimetic	Sigma-Aldrich/ PZ-0003

Imatinib 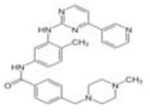	Tyrosin-kinase inhibitor used in the treatment of multiple cancers; inihibits c-kit and PDGF-R (platelet-derived growth factor receptor) signaling	Peptide mimetic	Selleckchem/ S1026
Sulfadiazine 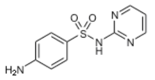	Sulfonamide antibiotic; stops the production of folic acid in parasites	Benzene sulfonamide	Sigma-Aldrich/ S8626
Amiodarone 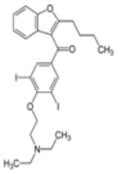	Class III of antiarrhythmic agents; blocks sodium channels	Tertiary amine	Sigma-Aldrich/ A8123
Chlorpromazine 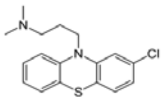	Dopamine antagonist possessing additional antiadrenergic, antiserotonergic, antichol inergic and antihistaminergic properties used to treat schizophrenia	Tertiary amine	Sigma-Aldrich/ C8138
Oxytocin 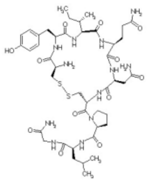	Peptide hormone; stimulates uterine contraction and lactation	Peptide/ Protein	R&D Systems/ 1910

Supplemental Material, Figure S2:
Chemical and pharmacological characteristics of the group of environmental pollutants

Compound	Pharmacological characteristics	Chemical characteristics	Developmental toxicity/Neurotoxicity evidences		Supplier info
			In vitro	In vivo	
Methoxyacetic acid 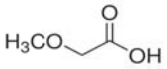	Phthalate ester (DMEP) and methoxyethanol metabolite formed rapidly formed in vivo from industrial solvents	Carboxylic acid	(1) (2)	(3) (4) (5)	Sigma-Aldrich/ 194557
Cyproconazole 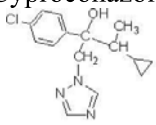	Pesticide, showing various teratogenic effects, via inhibition of cyp enzymesd and reduction of steroidogenesis	Triazole	(6) (7) (8)	(9)	Sigma-Aldrich/ 46068
Triadimefon 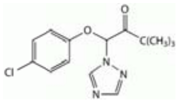	Pesticide; various teratogenic effects observed in rats	Triazole	(10) (11)	(12) (13)	Bayer Crop Science
PCB-153 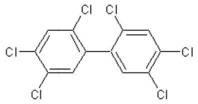	Non-planar polychlorinated biphenyl (environmental toxicant); acts on Ca^{2+} homeostasis and is teratogenic/ neurotoxic	Polychlorinated biphenyl	(14)	(15) (16)	
PBDE-99 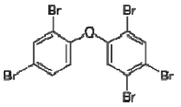	Flame retardant which belongs to the group of polybrominated diphenyl ethers (PBDEs); acts on Ca^{2+} homeostasis	Polybrominated diphenyl ether	(17) (18) (19)	(20) (21) (22) (23) (24)	
Arsenic trioxide 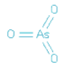	Targets cellular SH groups, and has multiple toxic effects; used for the treatment of certain leukemias.	Arsenite (As_2O_3)	(25)	(26)	Sigma-Aldrich/ 11099

(1) Daston et al. (1991); (2) Robinson et al. (2010); (3) Hermsen et al. (2011); (4) Scott et al. (1989); (5) Welsch et al. (2005); (6) Robinson et al. (2012); (7) Theunissen et al. (2012); (8) Heusinkveld et al. (2013); (9) Machera (1995); (10) Di Renzo et al. (2011a); (11) Zimmer et al. (2012); (12) Menegola et al. (2005); (13) Di Renzo et al. (2011b); (14) Johansson et al. (2006); (15) Piedrafita et al. (2008); (16) He P et al. (2011); (17) Madia et al. (2004); (18) Schreiber et al. (2010); (19) Alm et al. (2010); (20) Darnerud (2008); (21) Costa et al. (2008); (22) Eriksson et al. (2002); (23) Eriksson et al. (2006); (24) Branchi et al. (2005); (25) Vahidnia et al. (2007); (26) Golub et al. (1998)

Supplemental material, Figure S3:
PBPK modeling for the polybrominated diphenyl ethers PBDE-99

A

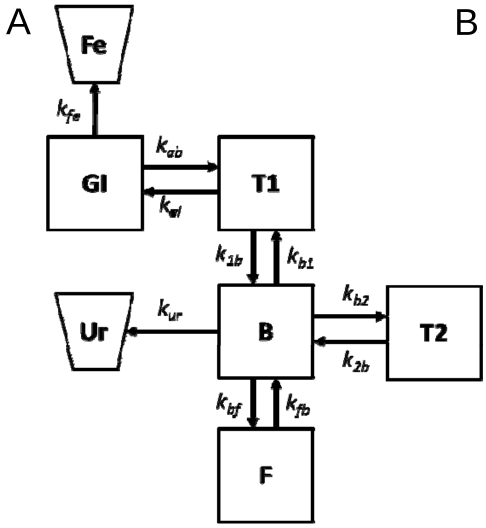

B

Rate constant		Value (h^{-1})
k_{ab}	absorption	0.7
k_{fe}	fecal excretion	0.13
k_{ur}	urinary excretion	0.03
k_{bf}	blood-to-fat transfer	0.65
k_{fb}	fat-to-blood transfer	0.006
k_{1b}	tissue1-to-blood transfer	0.5
k_{b1}	blood-to-tissue1 transfer	5
k_{2b}	tissue2-to-blood transfer	0.008
k_{b2}	blood-to-tissue2 transfer	1.1
k_{el}	elimination	0.28

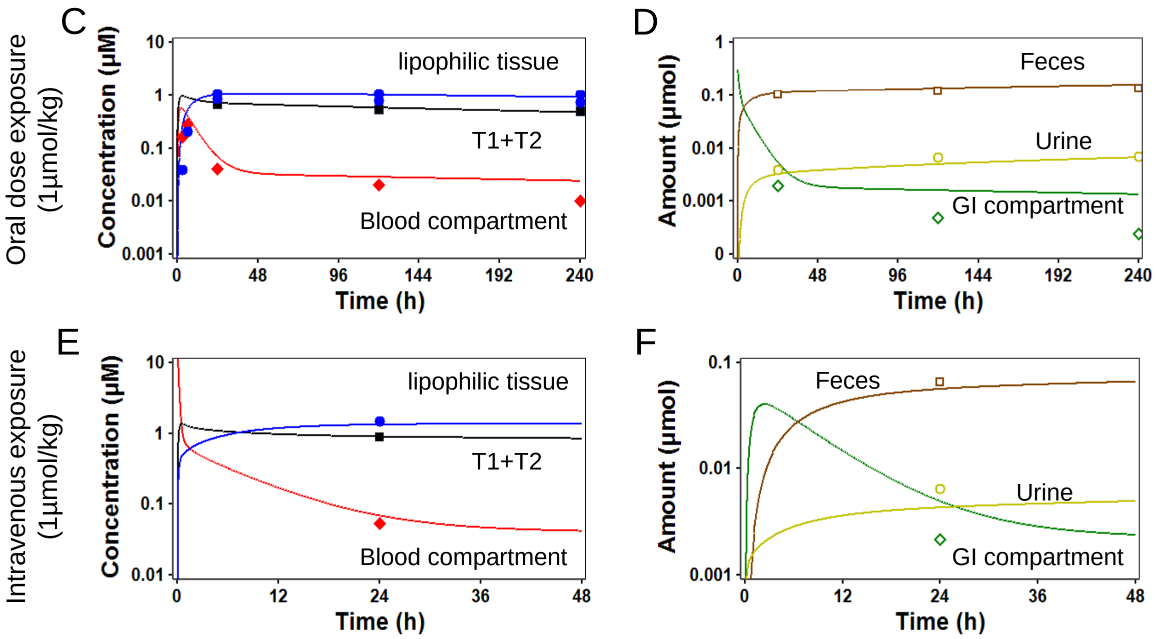

Supplemental material, Figure S3:
a Schematic representation of the PBPK model for PBDE-99, which was constructed based on data on tissue distribution, metabolism and excretion of PBDE-99 as described by Hakk et al. (2002) and Chen et al. (2006). The model contains a gastrointestinal lumen compartment (GI), two rapid equilibrium compartments (T1 and T2), a blood compartment (B), a lipophilic tissues compartment (F) representing adipose tissue and skin, and compartments for urinary and fecal excretion (Ur and Fe). **b** The exchange between blood and tissue compartments is described by first order rate constants which are reported with their parameter estimates. **c-f** Comparison between the PBPK model simulations and the values reported from Chen et al. (2006) is shown: upper panels show simulated (curves) and observed (symbols) **c** concentrations of PBDE-99 after a single oral dose of 1 µmol/kg, in blood, lipophilic and rapid equilibrium compartments and **d** the simulated and observed amounts of PBDE-99 in gut, feces and urine. Lower panels show simulated and observed **e** concentrations and **f** amounts of PBDE-99 after an intravenous dose of 1 µmol/kg, in blood, lipophilic and rapid equilibrium compartments and in gut, feces and urine, respectively.

Supplemental Material, Figure S4:
Information about clinical concentration ranges and free concentration ranges for chemicals belonging to the test battery compound list

For each compound tested in the test battery, clinical and epidemiological studies were chosen in order to determine a realistic clinical concentration range (CC) of the substance (total drug concentration in plasma). The free concentration range (FCC) was then calculated based on the plasma protein binding value. In case of environmental pollutants, it was not always possible to obtain data from human exposure studies; in these cases, in vivo and in vitro animal studies reporting developmental toxicity effects have been used to calculate the exposure range of interest.

Teriflunomide

MW: 270	CC: 10.8 µM	FCC: 54.0 nM

In Parekh et al. (2010), the mean pharmacokinetic parameters of teriflunomide are calculated following oral administration of 20 mg leflunomide tablet formulation in 12 healthy human subjects. The clinical concentration range extrapolated from the average maximal concentration (C_{max}) observed was of 2543 – 3299 ng/ml (9.4 – 12.2 µM).
The pharmacologically active metabolite is reported being extensively bound to plasma proteins (>99.3), primarily to albumin, with almost constant portion (0.5%) of free teriflunomide.
Based on these data, the free concentration range calculated was of 12.7 – 16.5 ng/ml (47.0 – 61.1 nM).

Nintedanib (BIBF 1120)

MW: 539	CC: 74.2 nM	FCC: 70.8 nM

In Mross et al. (2010), sixty-one patients with advanced cancers received BIBF 1120 in successive cohorts. Twenty-five subjects received 50 to 450 mg once daily and 36 received 150 to 300 mg twice daily in 4-week treatment courses interspersed by 1 week of washout.
The clinical concentration range extrapolated from the average C_{max} observed was 30 - 50 ng/ml (55.6 – 92.8 nM).
The portion of free nintedanib in plasma is predicted being 95.5%.
Based on these data, the free concentration range calculated was 28.6 – 47.7 ng/ml (53.1 – 88.5 nM).

Telaprevir

MW: 681	CC: 5.1 µM	FCC: 1.6 µM

In a phase 1b study, Yamada et al. (2012) examine safety, tolerability, pharmacokinetics of telaprevir in 10 patients infected with hepatitis C virus genotype 1b with high viral load (> 5 log10 IU/mL) and receiving 750 mg telaprevir every 8 h for 12 weeks.
The clinical concentration range observed, basing on the C_{max} values, is: 2 – 5 µg/ml (2.9 – 7.3 µM).
Telaprevir binds primarily to alpha 1-acid glycoprotein and albumin, in a concentration dependent manner. The drug is approximately 59% to 76% bound to human plasma proteins (compound data sheet).
Based on these values, the free concentration range calculated was 0.6 – 1.6 µg/ml (0.9 – 2.3 µM).

Sitagliptin

MW: 407	CC: 1.9 µM	FCC: 1.2 µM

In Herman et al. (2006b), the pharmacodynamics, pharmacokinetics, and tolerability of sitagliptin are examined after administration of single oral doses (25 or 200 mg) in a cohort of 58 patients with type 2 diabetes, not exposed to anti-hyperglycemic agents.
The clinical concentration range calculated basing on observed C_{max} was: 1.3 – 2.6 µM.
The level of binding to plasma proteins is of 38%. as reported in the pharmacokinetic study by Herman et al. (2006a).
The free concentration range was calculated, basing on the reported values and was of 0.8 - 1.6 µM.

Abiraterone

MW: 349	CC: 647.3 nM	FCC: 7.8 nM

Abiraterone is a novel potent, selective, irreversible inhibitor of CYP 17a-hydroxylase/C17,20-lyase enzyme. In Gurav et al. (2012), metastatic castration-resistant prostate cancer patients were followed after oral administration of arbirateron acetate (4*250 mg tablet).
The clinical concentration range calculated basing on the observed C_{max} was of 48 - 404 ng/ml (137.5 – 1157.6 nM). The ART is highly bound (>98.8%) to plasma proteins.
The free concentration range calculated from the reported values was 0.6 – 4.8 ng/ml (1.7 – 13.9 nM).

Roflumilast

MW: 403	CC: 18.0 nM	FCC: 0.2 nM

In de Mey et al. (2011), healthy men are treated with 500 µg tablet roflumilast once daily, for 16 days. The steady-state plasma pharmacokinetics of roflumilast as well as pharmacodynamics is evaluated on day 11.
Basing on the C_{max} observed, the clinical concentration range is of 4.94 – 9.64 ng/ml (12.2 – 23.9 nM).
The plasma protein binding of Roflumilast is 99% (Pinner et al. 2012).
Using the reported values, the free concentration range was calculated and was 0.0494 – 0.0964 ng/ml (0.12 – 0.24 nM).

Exenatide

MW: 4187	CC: 44.7 nM	FCC: 0.3 nM

In the assessment report compiled by the European Medicines Agency (EMEA) for the evaluation of medicines for human use, the maximal recommended human dose is 1.8 mg/day, which gives rise to a C_{max} of 44.7 nM.
In the study of Plum et al. (2013), *in vitro* protein binding of the drug in human plasma is evaluated and estimated being of 99.4%.
Based on these evidences, the calculated free concentration range was of 0.3 nM.

Gefitinib

MW: 446	CC: 1.2 µM	FCC: 34.0 nM

In Scheffler et al. (2011), maximum plasma concentration of gefitinib is evaluated after administration of multiple oral doses of 250 mg in patients with solid tumors.
The clinical concentration range reported from the obtained C_{max} is 265 – 814 ng/ml (594.1 – 1825.1 nM). In whole blood from cancer patients, 2.8% of free drug is observed.
Based on these evidences, the free concentration range calculated was of 7.4 – 22.8 ng/ml (16.6 – 51 nM).

Rivaroxaban

MW: 435	CC: 689.6 nM	FCC: 41.4 nM

In Mueck et al. (2011), a population pharmacokinetic model is developed using plasma samples from patients with acute deep-vein thrombosis. The clinical relevant peak concentration range is 200 – 400 ng/ml (459.8 – 919.5 nM).
In Grillo et al. (2012), a PBPK model is developed to simulate rivaroxaban pharmacokinetics in young (20 – 45 years) or older (55 – 65 years) subjects with normal renal function, mild, moderate and severe renal impairment, with or without concomitant use of the combined P-glycoprotein and moderate CYP3A4 inhibitor, erythromycin. The unbound fraction in plasma calculated in this study is of 6%.
Based on these values, the free concentration range calculated was of 12 – 24 ng/ml (27.6 – 55.2 nM).

Aliskiren

MW: 551	CC: 326.5 nM	FCC: 166 nM

Aliskiren pharmacokinetic and pharmodynamic parameters have been analyzed in the research of Tapaninen et al. (2011). In a randomized crossover study, 100 mg of the antifungal drug itraconazole, a P-glycoprotein and CYP-3A4 inhibitor, or placebo is given to 11 healthy volunteers twice daily for 5 days. On day 3, they ingest a single 150-mg dose of aliskiren, a renin inhibitor used in the treatment of hypertension. The extrapolation of the results from the average C_{max} showed a clinical concentration range of: 60 – 300 ng/ml (109.0 – 544.5 nM).
Protein binding of Aliskiren is reported to be moderate, in the range of 47-51% (Vaidyanathan et al. 2008). The free concentration range calculated using the reported values was 30.6 – 153 ng/ml (55.5 – 277 nM).

Galnon

MW: 679	CC: n.a.	FCC: n.a.

Neuregulin

MW: 40000	CC: 6.3 µM	FCC: n.a.

In Moondra et al. (2009), the mean serum neuregulin-1β levels in 9 healthy men range from 32 to 473 ng/ml (0.8 nM – 11.8 µM).

Erythropoietin

MW: 34000	CC: 0.3 nM	FCC: n.a.

In Xuereb et al. (2011), level of recombinant human erythropoietin is analyzed in whole blood from anonymous healthy volunteers. The clinical concentration range calculated was of 0.1 – 0.5 nM.

Geldanamycin

MW: 616 (17-DMAG)	CC: 0.8 µM	FCC: 0.5 µM

In Kummar et al. (2010), phase I dose-escalation study is used to determine the toxicity and maximum tolerated dose of 17-dimethylaminoethylamino-17-demethoxygeldanamycin (17-DMAG), a geldanamycin derivative, administered on a twice weekly schedule in patients with advanced cancer.
The clinical concentration range was calculated from the C_{max} value and it was 225 – 773 ng/ml (0.4 – 1.2 µM).
In Egorin et al. (2002), mice or rats were exposed to 17-DMAG i.v. bolus doses of 33.3, 50, and 75 mg/kg in order to perform pharmacokinetic studies.
From this study the level of 17-DMAG bound to plasma proteins is calculated as 30 – 45%.
The free concentration range predicted by using the reported values was: 140.6 – 483.1 ng/ml (0.2 – 0.8 µM).

G-CSF

MW: 19600	CC: 1.3 pM	FCC: n.a.

Laske et al. (2009) show that G-CSF (granulocyte colony-stimulating factor) plasma concentrations range from 10-40 pg/ml (average ~20 pg/ml) in 50 patients affected of Alzheimer's disease, compared to 5-80 pg/ml (average ~28 pg/ml) in 50 healthy controls.
Clinically relevant concentration calculated from the reported evidences was of 25 pg/ml (1.3 pM).

Interferon-β

MW: 22500	CC: 0.4 pM – 7.5 nM	FCC: 0.4 pM – 7.5 nM

The therapeutic concentration of IFN-β depends on the indication. Yung et al. (1991) demonstrate activity of IFN-β in patients with recurrent malignant glioma upon intravenous dose of 90 MIU three times per week, increasing the dose to 180 MIU after two weeks. Neurotoxicity is dose-limiting, with adverse events noted at and above this active dosing pattern.
Kappos et al. (2004) recommend treatment with 8 MIU subcutaneous on alternating days for patients affected by multiple sclerosis (MS). Though not significant, a higher rate of spontaneous abortion is noted in presence of *in utero* IFN-β exposure. Similar effects during pregnancy are also noted in cynomolgus monkey in an unpublished study reported by the US FDA (1999). In this study pregnant cynomolgus monkeys exposed to intramuscular doses of 0.2 MIU or 0.033 nmol IFN-β/kg/day from gestation day 90 through term show an increase in spontaneous abortions and/or fetal loss. Although the effects show no apparent dose-response, they can be considered treatment-related in view of the reported abortifacient effects of other interferons.
We estimated the concentrations corresponding to these clinical and/or neurotoxic IFN-β1a doses using the PK-PD models for cynomolgus monkey and human published by Mager et al. (Mager and Jusko 2002, Mager et al. 2003). Simulated exposure to intravenous dose reported to be used in glioma patients by Yung et al. (1991) lead to a maximum free IFN-β plasma concentration of 7.5 nM.

Simulation of the MS therapeutic dosing regimen of s.c. 8 MIU- IFN- β resulted in a maximum free plasma concentrations of 0.44 pM. Simulation of the subcutaneous dose of 0.033 nmol IFN-β/kg/day from GD 90 to term (GD 160) related to spontaneous abortions resulted in maximum plasma concentrations of IFN-β increasing from 1.9 on the first day to 3 pM at term.
Since aspecific binding is considered negligible, the free concentration range was considered corresponding to the clinical one.

Sildenafil

MW: 475	CC: 221.0 nM	FCC: 11.0 nM

In the study of Vachiery et al. (2011), the pharmacokinetics and pharmacodynamics of a 10 mg intravenous sildenafil bolus is assessed in pulmonary arterial hypertension patients. The clinical concentration range observed is 30 – 180 ng/ml (63.1 – 378.9 nM).
In Purvis et al. (2002), a total of 17 healthy male volunteers are randomized to receive a single 100 mg dose of sildenafil for two periods and a single dose of placebo for two periods, with each period separated by a minimum of 5 – 7 days. Blood samples are collected before each dose and at different time points after dose for measurement of sildenafil and metabolite concentrations. In this study the plasma protein binding is reported being as approximately 95%.
Using the reported values, the calculated free concentration range was of 1.5 – 9.0 ng/ml (3.2 – 18.9 nM).

Imatinib

MW: 494	CC: 3.0 µM	FCC: 224.0 nM

In Di Gion et al. (2011), the reported clinical concentration range is 1000 – 2000 ng/ml (2.0 – 4.0 µM).
In Kretz et al. (2004) the blood distribution and protein binding of imatinib are determined *in vitro* using ^{14}C labelled compounds. Blood samples are taken from healthy males exposed to 300-500 ng/ml, 5000 ng/ml, 12000 ng/ml and 26000 ng/ml of imatinib. The average level of unbound fraction of compound in plasma calculated is 7.4 %.
The free concentration range calculated using the reported values was of 74 – 148 ng/ml (149.7 – 299.6 nM).

Oxytocin

MW: 1007	CC: 2.0 nM	FCC: 2.0 nM

Evaluating different studies (Opacka-Juffry and Mohiyeddini 2012; Pierrehumbert et al. 2010; Scantamburlo et al. 2007), the average clinical concentration range was estimated as 10 – 4000 pg/ml (0.01 – 4.0 nM).
In Fabian et al. (1969) study, it is stated that oxytocin in plasma remains unbound.
The free concentration range calculated basing on the reported studies was 0.01 – 4.0 nM (2.0 nM).

Sulfadiazine

MW: 250	CC: 320.0 µM	FCC: 160.0 µM

In Jordan et al. (2004), the pharmacokinetics of a dose of 2000 mg of sulfadiazine administered twice daily versus those of 1000 mg administered four times every day are compared in eight human immunodeficiency virus-infected patients. Serial blood samples are collected following administration of the morning dose on the fifth day after the initiation of each new regimen. Plasma samples ae collected over 48 h and assayed by a validated high-performance liquid chromatography method.
The average C_{max} showed a clinical concentration range of 50 – 110 µg/ml (200.0 – 440.0 µM).
In Mannisto et al. (1982), the pharmacokinetic of sulphadiazine/trimethoprim (1000 mg/320 mg) is evaluated after intravenous infusion of the drugs over a 60 min period to six young, healthy volunteers. In this study the degree of protein binding is evaluated as 50%.
Based on the reported studies, the free concentration range calculated was 25 – 55 µg/ml (100.0 – 220.0 µM).

Amiodarone

MW: 645	CC: 2.3 µM	FCC: 0.5 nM

In the study of Shiga et al. (2011), thirty-two healthy Japanese male volunteers (20–32 years) are randomized to three single-dose groups (1.25, 2.5 and 5.0 mg/kg) of intravenous amiodarone. The average value of plasma concentration range is of 1 – 2 µg/ml (1.5 – 3.1 µM).
In Veronese et al. (1988), the plasma protein binding of amiodarone is measured by erythrocyte partitioning, and found to be the same in six healthy subjects and eight patients being treated for cardiac arrhythmias; the average value is of 99.98%.
Using the values reported from these studies, the free concentration range calculated was 0.2 – 0.4 ng/ml (0.31 – 0.62 nM).

Chlorpromazine

MW: 318	CC: 393.1 nM	FCC: 58.9 nM

In Borges et al. (2011), the values of plasma concentration of chlorpromazine is evaluated in 72 healthy volunteers of both sexes aged between 18-50 years, exposed to a single oral dose of 100 mg chlorpromazine. The clinically relevant concentration reported is of 100 – 150 ng/ml (314.5 – 471.7 nM).
In Yeung et al. (1993), the pharmacokinetics of chlorpromazine is investigated in 11 healthy young men after a bolus intravenous (i.v.) dose (10 mg) and three single oral doses (25, 50 and 100 mg), with a washout period of two weeks between doses. The drug is revealed being bound to the plasma protein albumin with a percentage around 85%.
The free concentration range calculated from the reported values was 15 – 22.5 ng/ml (47.2 – 70.7 nM).

Methoxyacetic acid

MW: 90	Human exposure concentration: 60 µM	DT concentration: 7.5 mM

Methoxyacetic acid (MAA) is the major metabolite of ethyl glycol monomethyl ether (EGME) a compound, widely used in the working environment (Henley and Korach 2010). Like valproic acid, chemically related to MAA, it inhibits histone deacetylates and alters gene expression via histone hyperacetylation. MAA is an endocrine disruptor and supposed to be responsible for the reproductive toxicity of EGME (Foster et al. 1984). The mode of action is mainly via perturbations of estrogen signaling. a mechanism that could be used to link this compound to DNT. Estimation of human blood concentration for MAA due to EGME permitted occupational exposure is approximately 60 µM (Welsch 2005). PBPK simulation of *in vivo* adverse effects doses (in rats and mice) show a maternal plasma MAA maximal concentration of 6 – 9 mM (Welsch 2005).
In vitro studies confirm this concentration range; in Piersma et al. (2008), the BMC_5 of MAA in the whole embryo culture morphological assay is reported being of 1.6 mM, while the BMC_{50} in the embryonic stem cell differentiation assay of the Embryonic Stem cell Test (EST) is of 2.4 mM (de Jong et al. 2009).

Cyproconazole

MW: 292	Human exposure concentration: n.a.	DT concentration: 65-118 µM

Cyproconazole is an azole fungicide that inhibits ergosterol biosynthesis in fungi. The toxic mode of action of azole fungicides in mainly based on the inhibition of mammalian cytochrome P450 enzymes with a possible impact on retinoic acid (RA) concentration. No human data are available on teratogenic effects of cyproconazole (Giavini and Menegola 2010). In a rat study, cleft palate and hydrocepathy are identified as the most common malformations of the fetuses exposed to the fungicide, with a suggested NOEL of 20 mg/kg (Machera 1995). In an *in vivo* study by Hermsen et al. (2012), the exposure of zebrafish embryos to 65 µM cyproconazole show an increase in teratogenic effects such as pericardial edema and malformations of head and heart. In Robinson et al. (2012), the teratogenic effects of cyproconazole are showed in rat whole embryo culture; the exposure to concentrations of 1 and 1.7 mM for 48 hours leads, respectively, to 50% and the 100% of malformations. In another *in vitro* study by Heusinkveld et al. (2013), the neurotoxicity of cyproconazole is analyzed in dopaminergic PC12 cells by cell viability and intracellular calcium concentration level analysis; the inhibitory concentration of the azole fungicide on depolarization-evoked intracellular calcium concentration is 65 µM. In an *in vitro* study by Theunissen et al. (2012), neural embryonic stem cell test (ESTn) is used to evaluate the transcriptomic concentration-response to cyproconazole; concentration reducing cell viability to 80% is of 117.6 µM; the fungicide also showed to regulate genes within the neuron development GO-term (dendrite microtubule formation, early ectodermal development transcription factors).

Triadimefon

MW: 293.75	Human exposure concentration: n.a.	DT concentration: 27 – 500 µM

Triadimefon is a fungicide belonging to the conazole class, together with cyproconazole and its metabolite triadimenol. These pesticides are known to disturb steroid homeostasis in mammals (Crowell et al. 2011). A range of toxic effects is observed in rats at doses around 50 mg/kg/day, including effects on male fertility and CNS toxicity. Goetz et al. (2007) report increased testosterone levels and related effects (reduced testes weight, increased anogenital distance) in male offspring of Wistar rats perinatally exposed to 47 mg/kg/day.
Crofton et al. (2011) report that adult male Long-Evans rats exposed to a single dose of 50 mg/kg triadimefon or triadimenol by oral gavage show transient CNS toxicity as indicated by hyperactivity and stereotyped behavior. The same neurotoxic symptoms are observed upon chronic dietary exposure of male Wistar rats to 54.6 mg/kg/day (US EPA (2006)). Our simulation of this exposure suggested a corresponding central nervous system C_{max} of 27 µM and 196 µM (for dietary and acute exposure, respectively) (see in the text). Di Renzo et al. (2011) observe the induction of abnormalities (craniofacial defects, bent forebrain and abnormal hindbrain segmentation) in Xenopus laevis embryos treated during early neurulation phases with 500 µM triadimefon. In vitro studies in rat embryos confirmed this toxic concentration range (Menegola et al. 2000, Di Renzo et al. 2009).

PCB-153

MW: 361	Human exposure concentration: 0.3 –1.8 nM	DT concentration: 388 pM

The range of human exposure to the PCB-153 is showed by Covaci et al. (2002) where the level of PCB-153 in maternal serum concentrations of 670 ± 350 pg/ml (0.3 - 1.8 nM).
In a study by Govarts et al. (2012), the PCB-153 concentration in maternal and cord blood is measured in a cohort of 7990 women. The median concentration of cords serum PCB-153 is 140 ng/l (388 pM). Linear regression of birth weight on estimated of cord serum concentrations show that low-level exposure to PCB impairs fetal growth, with an inverse association corresponding to a 150 g reduction per 1 µg/l increase in cord serum PCB-153.

PBDE-99

MW: 568	Human exposure concentration: 5 – 97 pM	DT concentration: 0.1 – 1.3 µM

The toxicity of PBDE-99 is reviewed by US EPA in 2008 (US EPA (2008)); this document collects diverse epidemiological studies, reporting the PBDE-99 concentration in different human biological samples. The concentration range in serum is between 0.6 ng/g lw and 11 ng/g lw. Under the assumption that there is 0.5% lipid in blood, total blood concentrations can be calculated; the resulting concentration range was between 3 ng/l and 55 ng/l (5 pM- 97 pM).
In the study by Eskenazi et al. (2013), the combined effect of PBDEs on neurobehavioral readouts is investigated; especially high exposed children show pronounced deficits. The geometric mean of PBDE-99 concentration in maternal serum is of 4.5 ng/g, corresponding to 25 ng/l (44 pM). After PBDE-47, this congener yields the highest concentration in maternal serum and in child serum samples collected at 7 years of age. In Gascon et al. (2012), PBDE-99 concentrations in breast milk samples are provided; the average concentration is 0.27 ng/g lw, corresponding to 1 ng/l (1.8 pM). In

both human developmental neurotoxicity studies, the behavioral effects are caused by mixed exposures and the specific contribution of this congener to the effects is unclear
A simpler exposure situation has been reproduced in *in vivo* studies; effects on neurodevelopment and fertility in adult rodents after perinatal exposure to low doses of PBDE-99 have been observed. Kuriyama et al. (2005) find hyperactivity and impaired spermatogenesis in Wistar rat offspring after exposure of the dams to a single oral dose of 0.11 µmol/kg on gestational day 6. Viberg et al. (2005) observe effects on spontaneous behavior of Sprague-Dawley rats exposed to doses between 1.4 and 14 µmol/kg on postnatal day 10. The simulation of this exposure suggested a corresponding C_{max} of 0.10 µM and 1.3 µM (respectively for the two studies) in the rapid equilibrium compartment (see in the text).

Arsenic Trioxide

MW: 198	Human exposure concentration: 0.2 –1.1 µM	DT concentration: 0.2 – 1.1 µM

Many case studies on arsenic poisoning have been reported, but they include the contribution of mixtures of various inorganic arsenic compounds. Inorganic arsenic compounds differ markedly with respect to their water solubility and arsenic trioxide belongs to the more water-soluble compounds, normally associated to a lower accumulation in soft tissues.
Arsenic is also used for its therapeutic effects in the treatment of acute promyielocytic leukaemia (APL); as side effects, many APL patients show development of neuropatological syntoms after injection with arsenic trioxide (up to 0.15 µg/kg daily) (Vahidnia et al. 2008). The toxicity mode of action of this drug is still unknown; in an *in vivo* study in rats, induction of axonal degeneration has been hypothesized as a possible toxicity-inducing mechanism (Vahidnia et al. 2008).
In the study by Hua et al. (2011), 10 mg arsenic trioxide is administered intravenously over a 4 hour period to Chinese primary hepatocarcinoma patients. Arsenic peak plasma concentrations are reported to be 47 – 225.8 ng/ml; (237.0 nM - 1140.4 nM). These doses have also been reported to be toxic. However, the therapeutic effect (anticancer) outweighs the toxic side effects, to a certain extent (Au and Kwong 2008). Zhang et al. (1998) show a ratio of 5.6% of total arsenic bound to serum proteins. Free plasma protein concentration range can be calculated as 233.7 – 1078.8 nM.

Supplemental material, Figure S5:
Simulation of toxic exposure to PBDE-99 by a PBPK modeling approach

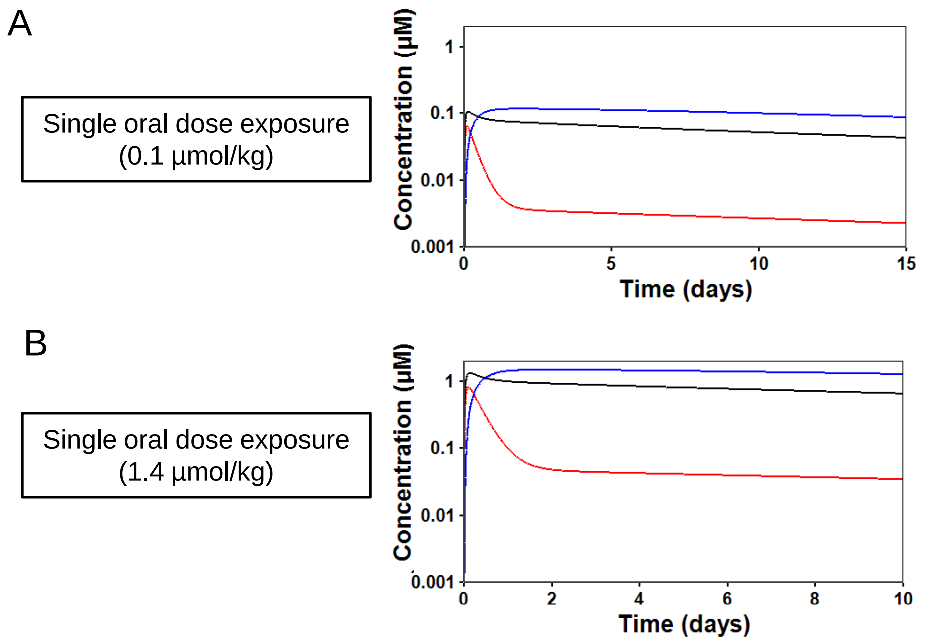

Supplemental material, Figure S5: Simulation of PBDE concentration in blood (red line), lipophilic (blue line) and rapid equilibrium (black line) compartment induced by a single oral dose of **a** 0.1 µmol/kg or **b** 1.4 µmol/kg in rats, using the PBPK model described in Fig. S3.

Supplemental Bibliography

Alm H, Scholz B, Kultima K, Nilsson A, Andren PE, Savitski MM, et al. (2010) In vitro neurotoxicity of PBDE-99: immediate and concentration-dependent effects on protein expression in cerebral cortex cells. Journal of proteome research 9(3):1226-35 doi:10.1021/pr900723c

Au WY, Kwong YL (2008) Arsenic trioxide: safety issues and their management. Acta pharmacologica Sinica 29(3):296-304 doi:10.1111/j.1745-7254.2008.00771.x

Borges NC, Rezende VM, Santana JM, Moreira RP, Moreira RF, Moreno P, et al. (2011) Chlorpromazine quantification in human plasma by UPLC-electrospray ionization tandem mass spectrometry. Application to a comparative pharmacokinetic study. Journal of chromatography B, Analytical technologies in the biomedical and life sciences 879(31):3728-34 doi:10.1016/j.jchromb.2011.10.020

Branchi I, Capone F, Vitalone A, Madia F, Santucci D, Alleva E, et al. (2005) Early developmental exposure to BDE 99 or Aroclor 1254 affects neurobehavioural profile: interference from the administration route. Neurotoxicology 26(2):183-92 doi:10.1016/j.neuro.2004.11.005

Chen LJ, Lebetkin EH, Sanders JM, Burka LT (2006) Metabolism and disposition of 2,2',4,4',5-pentabromodiphenyl ether (BDE99) following a single or repeated administration to rats or mice. Xenobiotica; the fate of foreign compounds in biological systems 36(6):515-34 doi:10.1080/00498250600674477

Costa LG, Giordano G, Tagliaferri S, Caglieri A, Mutti A (2008) Polybrominated diphenyl ether (PBDE) flame retardants: environmental contamination, human body burden and potential adverse health effects. Acta bio-medica : Atenei Parmensis 79(3):172-83

Covaci A, Jorens P, Jacquemyn Y, Schepens P (2002) Distribution of PCBs and organochlorine pesticides in umbilical cord and maternal serum. The Science of the total environment 298(1-3):45-53

Crofton KM, Mundy WR, Lein PJ, Bal-Price A, Coecke S, Seiler AE, et al. (2011) Developmental neurotoxicity testing: recommendations for developing alternative methods for the screening and prioritization of chemicals. Altex 28(1):9-15

Crowell SR, Henderson WM, Kenneke JF, Fisher JW (2011) Development and application of a physiologically based pharmacokinetic model for triadimefon and its metabolite triadimenol in rats and humans. Toxicology letters 205(2):154-62 doi:10.1016/j.toxlet.2011.05.1036

Darnerud PO (2008) Brominated flame retardants as possible endocrine disrupters. International journal of andrology 31(2):152-60 doi:10.1111/j.1365-2605.2008.00869.x

Daston GP, Baines D, Yonker JE (1991) Chick embryo neural retina cell culture as a screen for developmental toxicity. Toxicology and applied pharmacology 109(2):352-66

de Jong E, Louisse J, Verwei M, Blaauboer BJ, van de Sandt JJ, Woutersen RA, et al. (2009) Relative developmental toxicity of glycol ether alkoxy acid metabolites in the embryonic stem cell test as compared with the in vivo potency of their parent compounds. Toxicological sciences : an official journal of the Society of Toxicology 110(1):117-24 doi:10.1093/toxsci/kfp083

de Mey C, Nassr N, Lahu G (2011) No relevant cardiac, pharmacokinetic or safety interactions between roflumilast and inhaled formoterol in healthy subjects: an open-label, randomised, actively controlled study. BMC clinical pharmacology 11:7 doi:10.1186/1472-6904-11-7

Di Gion P, Kanefendt F, Lindauer A, Scheffler M, Doroshyenko O, Fuhr U, et al. (2011) Clinical pharmacokinetics of tyrosine kinase inhibitors: focus on pyrimidines, pyridines and pyrroles. Clinical pharmacokinetics 50(9):551-603 doi:10.2165/11593320-000000000-00000

Di Renzo F, Bacchetta R, Sangiorgio L, Bizzo A, Menegola E (2011a) The agrochemical fungicide triadimefon induces abnormalities in Xenopus laevis embryos. Reprod Toxicol 31(4):486-93 doi:10.1016/j.reprotox.2011.01.003

Di Renzo F, Corsini E, Broccia ML, Marinovich M, Galli CL, Giavini E, et al. (2009) Molecular mechanism of teratogenic effects induced by the fungicide triadimefon: study of the expression of TGF-beta mRNA and TGF-beta and CRABPI proteins during rat in vitro development. Toxicology and applied pharmacology 234(1):107-16 doi:10.1016/j.taap.2008.09.025

Di Renzo F, Rossi F, Prati M, Giavini E, Menegola E (2011b) Early genetic control of craniofacial development is affected by the in vitro exposure of rat embryos to the fungicide triadimefon.

Birth defects research Part B, Developmental and reproductive toxicology 92(1):77-81 doi:10.1002/bdrb.20284
Egorin MJ, Lagattuta TF, Hamburger DR, Covey JM, White KD, Musser SM, et al. (2002) Pharmacokinetics, tissue distribution, and metabolism of 17-(dimethylaminoethylamino)-17-demethoxygeldanamycin (NSC 707545) in CD2F1 mice and Fischer 344 rats. Cancer chemotherapy and pharmacology 49(1):7-19
Eriksson P, Fischer C, Fredriksson A (2006) Polybrominated diphenyl ethers, a group of brominated flame retardants, can interact with polychlorinated biphenyls in enhancing developmental neurobehavioral defects. Toxicological sciences : an official journal of the Society of Toxicology 94(2):302-9 doi:10.1093/toxsci/kfl109
Eriksson P, Viberg H, Jakobsson E, Orn U, Fredriksson A (2002) A brominated flame retardant, 2,2',4,4',5-pentabromodiphenyl ether: uptake, retention, and induction of neurobehavioral alterations in mice during a critical phase of neonatal brain development. Toxicological sciences : an official journal of the Society of Toxicology 67(1):98-103
Eskenazi B, Chevrier J, Rauch SA, Kogut K, Harley KG, Johnson C, et al. (2013) In utero and childhood polybrominated diphenyl ether (PBDE) exposures and neurodevelopment in the CHAMACOS study. Environ Health Perspect 121(2):257-62 doi:10.1289/ehp.1205597
Fabian M, Forsling ML, Jones JJ, Lee J (1969) The release, clearance and plasma protein binding of oxytocin in the anaesthetized rat. The Journal of endocrinology 43(2):175-89
FDA toxicologist’s review of interferon-β 1a. http://www.fda.gov/downloads/Drugs/DevelopmentApprovalProcess/HowDrugsareDevelopedandApproved/ApprovalApplications/TherapeuticBiologicApplications/ucm106138.pdf
Foster PM, Creasy DM, Foster JR, Gray TJ (1984) Testicular toxicity produced by ethylene glycol monomethyl and monoethyl ethers in the rat. Environ Health Perspect 57:207-17
Gascon M, Fort M, Martinez D, Carsin AE, Forns J, Grimalt JO, et al. (2012) Polybrominated diphenyl ethers (PBDEs) in breast milk and neuropsychological development in infants. Environ Health Perspect 120(12):1760-5 doi:10.1289/ehp.1205266
Giavini E, Menegola E (2010) Are azole fungicides a teratogenic risk for human conceptus? Toxicol Lett 198(2):106-11 doi:10.1016/j.toxlet.2010.07.005
Goetz AK, Ren H, Schmid JE, Blystone CR, Thillainadarajah I, Best DS, et al. (2007) Disruption of testosterone homeostasis as a mode of action for the reproductive toxicity of triazole fungicides in the male rat. Toxicological sciences : an official journal of the Society of Toxicology 95(1):227-39 doi:10.1093/toxsci/kfl124
Golub MS, Macintosh MS, Baumrind N (1998) Developmental and reproductive toxicity of inorganic arsenic: animal studies and human concerns. Journal of toxicology and environmental health Part B, Critical reviews 1(3):199-241 doi:10.1080/10937409809524552
Govarts E, Nieuwenhuijsen M, Schoeters G, Ballester F, Bloemen K, de Boer M, et al. (2012) Birth weight and prenatal exposure to polychlorinated biphenyls (PCBs) and dichlorodiphenyldichloroethylene (DDE): a meta-analysis within 12 European Birth Cohorts. Environmental health perspectives 120(2):162-70 doi:10.1289/ehp.1103767
Grillo JA, Zhao P, Bullock J, Booth BP, Lu M, Robie-Suh K, et al. (2012) Utility of a physiologically-based pharmacokinetic (PBPK) modeling approach to quantitatively predict a complex drug-drug-disease interaction scenario for rivaroxaban during the drug review process: implications for clinical practice. Biopharmaceutics & drug disposition 33(2):99-110 doi:10.1002/bdd.1771
Gurav S, Punde R, Farooqui J, Zainuddin M, Rajagopal S, Mullangi R (2012) Development and validation of a highly sensitive method for the determination of abiraterone in rat and human plasma by LC-MS/MS-ESI: application to a pharmacokinetic study. Biomedical chromatography : BMC 26(6):761-8 doi:10.1002/bmc.1726
Hakk H, Larsen G, Klasson-Wehler E (2002) Tissue disposition, excretion and metabolism of 2,2',4,4',5-pentabromodiphenyl ether (BDE-99) in the male Sprague-Dawley rat. Xenobiotica; the fate of foreign compounds in biological systems 32(5):369-82 doi:10.1080/00498250110119117
He P, Wang A, Niu Q, Guo L, Xia T, Chen X (2011) Toxic effect of PBDE-47 on thyroid development, learning, and memory, and the interaction between PBDE-47 and PCB153 that

enhances toxicity in rats. Toxicology and industrial health 27(3):279-88 doi:10.1177/0748233710387002

Henley DV, Korach KS (2010) Physiological effects and mechanisms of action of endocrine disrupting chemicals that alter estrogen signaling. Hormones 9(3):191-205

Herman GA, Bergman A, Liu F, Stevens C, Wang AQ, Zeng W, et al. (2006a) Pharmacokinetics and pharmacodynamic effects of the oral DPP-4 inhibitor sitagliptin in middle-aged obese subjects. Journal of clinical pharmacology 46(8):876-86 doi:10.1177/0091270006289850

Herman GA, Bergman A, Stevens C, Kotey P, Yi B, Zhao P, et al. (2006b) Effect of single oral doses of sitagliptin, a dipeptidyl peptidase-4 inhibitor, on incretin and plasma glucose levels after an oral glucose tolerance test in patients with type 2 diabetes. The Journal of clinical endocrinology and metabolism 91(11):4612-9 doi:10.1210/jc.2006-1009

Hermsen SA, Pronk TE, van den Brandhof EJ, van der Ven LT, Piersma AH (2012) Triazole-induced gene expression changes in the zebrafish embryo. Reprod Toxicol 34(2):216-24 doi:10.1016/j.reprotox.2012.05.093

Hermsen SA, van den Brandhof EJ, van der Ven LT, Piersma AH (2011) Relative embryotoxicity of two classes of chemicals in a modified zebrafish embryotoxicity test and comparison with their in vivo potencies. Toxicology in vitro : an international journal published in association with BIBRA 25(3):745-53 doi:10.1016/j.tiv.2011.01.005

Heusinkveld HJ, Molendijk J, van den Berg M, Westerink RH (2013a) Azole fungicides disturb intracellular Ca2+ in an additive manner in dopaminergic PC12 cells. Toxicological sciences : an official journal of the Society of Toxicology 134(2):374-81 doi:10.1093/toxsci/kft119

Heusinkveld HJ, Molendijk J, van den Berg M, Westerink RH (2013b) Azole Fungicides Disturb Intracellular Ca2+ in an Additive Manner in Dopaminergic PC12 Cells. Toxicological sciences : an official journal of the Society of Toxicology doi:10.1093/toxsci/kft119

Hua H, Qin S, Rui J, Li J (2011) Pharmacokinetics of arsenic trioxide (As(2)O(3)) in Chinese primary hepatocarcinoma patients. Asian Pacific journal of cancer prevention : APJCP 12(1):61-5

Johansson C, Tofighi R, Tamm C, Goldoni M, Mutti A, Ceccatelli S (2006) Cell death mechanisms in AtT20 pituitary cells exposed to polychlorinated biphenyls (PCB 126 and PCB 153) and methylmercury. Toxicology letters 167(3):183-90 doi:10.1016/j.toxlet.2006.09.006

Jordan MK, Burstein AH, Rock-Kress D, Alfaro RM, Pau AK, Kovacs JA, et al. (2004) Plasma pharmacokinetics of sulfadiazine administered twice daily versus four times daily are similar in human immunodeficiency virus-infected patients. Antimicrobial agents and chemotherapy 48(2):635-7

Kappos L, Weinshenker B, Pozzilli C, Thompson AJ, Dahlke F, Beckmann K, et al. (2004) Interferon beta-1b in secondary progressive MS: a combined analysis of the two trials. Neurology 63(10):1779-87

Kretz O, Weiss HM, Schumacher MM, Gross G (2004) In vitro blood distribution and plasma protein binding of the tyrosine kinase inhibitor imatinib and its active metabolite, CGP74588, in rat, mouse, dog, monkey, healthy humans and patients with acute lymphatic leukaemia. British journal of clinical pharmacology 58(2):212-6 doi:10.1111/j.1365-2125.2004.02117.x

Kummar S, Gutierrez ME, Gardner ER, Chen X, Figg WD, Zajac-Kaye M, et al. (2010) Phase I trial of 17-dimethylaminoethylamino-17-demethoxygeldanamycin (17-DMAG), a heat shock protein inhibitor, administered twice weekly in patients with advanced malignancies. Eur J Cancer 46(2):340-7 doi:10.1016/j.ejca.2009.10.026

Kuriyama SN, Talsness CE, Grote K, Chahoud I (2005) Developmental exposure to low dose PBDE 99: effects on male fertility and neurobehavior in rat offspring. Environmental health perspectives 113(2):149-54

Laske C, Stellos K, Stransky E, Leyhe T, Gawaz M (2009) Decreased plasma levels of granulocyte-colony stimulating factor (G-CSF) in patients with early Alzheimer's disease. Journal of Alzheimer's disease : JAD 17(1):115-23 doi:10.3233/JAD-2009-1017

Machera K (1995) Developmental toxicity of cyproconazole, an inhibitor of fungal ergosterol biosynthesis, in the rat. Bulletin of environmental contamination and toxicology 54(3):363-9

Madia F, Giordano G, Fattori V, Vitalone A, Branchi I, Capone F, et al. (2004) Differential in vitro neurotoxicity of the flame retardant PBDE-99 and of the PCB Aroclor 1254 in human astrocytoma cells. Toxicology letters 154(1-2):11-21 doi:10.1016/j.toxlet.2004.06.013

Mager DE, Jusko WJ (2002) Receptor-mediated pharmacokinetic/pharmacodynamic model of interferon-beta 1a in humans. Pharmaceutical research 19(10):1537-43

Mager DE, Neuteboom B, Efthymiopoulos C, Munafo A, Jusko WJ (2003) Receptor-mediated pharmacokinetics and pharmacodynamics of interferon-beta1a in monkeys. The Journal of pharmacology and experimental therapeutics 306(1):262-70 doi:10.1124/jpet.103.049502

Mannisto PT, Mantyla R, Mattila J, Nykanen S, Lamminsivu U (1982) Comparison of pharmacokinetics of sulphadiazine and sulphamethoxazole after intravenous infusion. The Journal of antimicrobial chemotherapy 9(6):461-70

Menegola E, Broccia ML, Di Renzo F, Massa V, Giavini E (2005) Craniofacial and axial skeletal defects induced by the fungicide triadimefon in the mouse. Birth defects research Part B, Developmental and reproductive toxicology 74(2):185-95 doi:10.1002/bdrb.20035

Menegola E, Broccia ML, Di Renzo F, Prati M, Giavini E (2000) In vitro teratogenic potential of two antifungal triazoles: triadimefon and triadimenol. In vitro cellular & developmental biology Animal 36(2):88-95 doi:10.1290/1071-2690(2000)036<0088:IVTPOT>2.0.CO;2

Moondra V, Sarma S, Buxton T, Safa R, Cote G, Storer T, et al. (2009) Serum Neuregulin-1beta as a Biomarker of Cardiovascular Fitness. The open biomarkers journal 2:1-5 doi:10.2174/1875318300902010001

Mross K, Stefanic M, Gmehling D, Frost A, Baas F, Unger C, et al. (2010) Phase I study of the angiogenesis inhibitor BIBF 1120 in patients with advanced solid tumors. Clinical cancer research : an official journal of the American Association for Cancer Research 16(1):311-9 doi:10.1158/1078-0432.CCR-09-0694

Mueck W, Lensing AW, Agnelli G, Decousus H, Prandoni P, Misselwitz F (2011) Rivaroxaban: population pharmacokinetic analyses in patients treated for acute deep-vein thrombosis and exposure simulations in patients with atrial fibrillation treated for stroke prevention. Clinical pharmacokinetics 50(10):675-86 doi:10.2165/11595320-000000000-00000

Opacka-Juffry J, Mohiyeddini C (2012) Experience of stress in childhood negatively correlates with plasma oxytocin concentration in adult men. Stress 15(1):1-10 doi:10.3109/10253890.2011.560309

Parekh JM, Vaghela RN, Sutariya DK, Sanyal M, Yadav M, Shrivastav PS (2010) Chromatographic separation and sensitive determination of teriflunomide, an active metabolite of leflunomide in human plasma by liquid chromatography-tandem mass spectrometry. Journal of chromatography B, Analytical technologies in the biomedical and life sciences 878(24):2217-25 doi:10.1016/j.jchromb.2010.06.028

Piedrafita B, Erceg S, Cauli O, Monfort P, Felipo V (2008) Developmental exposure to polychlorinated biphenyls PCB153 or PCB126 impairs learning ability in young but not in adult rats. The European journal of neuroscience 27(1):177-82 doi:10.1111/j.1460-9568.2007.5988.x

Pierrehumbert B, Torrisi R, Laufer D, Halfon O, Ansermet F, Beck Popovic M (2010) Oxytocin response to an experimental psychosocial challenge in adults exposed to traumatic experiences during childhood or adolescence. Neuroscience 166(1):168-77 doi:10.1016/j.neuroscience.2009.12.016

Piersma AH, Janer G, Wolterink G, Bessems JG, Hakkert BC, Slob W (2008) Quantitative extrapolation of in vitro whole embryo culture embryotoxicity data to developmental toxicity in vivo using the benchmark dose approach. Toxicological sciences : an official journal of the Society of Toxicology 101(1):91-100 doi:10.1093/toxsci/kfm253

Pinner NA, Hamilton LA, Hughes A (2012) Roflumilast: a phosphodiesterase-4 inhibitor for the treatment of severe chronic obstructive pulmonary disease. Clinical therapeutics 34(1):56-66 doi:10.1016/j.clinthera.2011.12.008

Plum A, Jensen LB, Kristensen JB (2013) In vitro protein binding of liraglutide in human plasma determined by reiterated stepwise equilibrium dialysis. Journal of pharmaceutical sciences 102(8):2882-8 doi:10.1002/jps.23648

Purvis K, Muirhead GJ, Harness JA (2002) The effects of sildenafil on human sperm function in healthy volunteers. British journal of clinical pharmacology 53 Suppl 1:53S-60S

Robinson JF, Tonk EC, Verhoef A, Piersma AH (2012) Triazole induced concentration-related gene signatures in rat whole embryo culture. Reprod Toxicol 34(2):275-83 doi:10.1016/j.reprotox.2012.05.088

Robinson JF, van Beelen VA, Verhoef A, Renkens MF, Luijten M, van Herwijnen MH, et al. (2010) Embryotoxicant-specific transcriptomic responses in rat postimplantation whole-embryo culture. Toxicological sciences : an official journal of the Society of Toxicology 118(2):675-85 doi:10.1093/toxsci/kfq292

Scantamburlo G, Hansenne M, Fuchs S, Pitchot W, Marechal P, Pequeux C, et al. (2007) Plasma oxytocin levels and anxiety in patients with major depression. Psychoneuroendocrinology 32(4):407-10 doi:10.1016/j.psyneuen.2007.01.009

Scheffler M, Di Gion P, Doroshyenko O, Wolf J, Fuhr U (2011) Clinical pharmacokinetics of tyrosine kinase inhibitors: focus on 4-anilinoquinazolines. Clinical pharmacokinetics 50(6):371-403 doi:10.2165/11587020-000000000-00000

Schreiber T, Gassmann K, Gotz C, Hubenthal U, Moors M, Krause G, et al. (2010) Polybrominated diphenyl ethers induce developmental neurotoxicity in a human in vitro model: evidence for endocrine disruption. Environmental health perspectives 118(4):572-8 doi:10.1289/ehp.0901435

Scott WJ, Fradkin R, Wittfoht W, Nau H (1989) Teratologic potential of 2-methoxyethanol and transplacental distribution of its metabolite, 2-methoxyacetic acid, in non-human primates. Teratology 39(4):363-73 doi:10.1002/tera.1420390408

Shiga T, Tanaka T, Irie S, Hagiwara N, Kasanuki H (2011) Pharmacokinetics of intravenous amiodarone and its electrocardiographic effects on healthy Japanese subjects. Heart and vessels 26(3):274-81 doi:10.1007/s00380-010-0047-7

Tapaninen T, Backman JT, Kurkinen KJ, Neuvonen PJ, Niemi M (2011) Itraconazole, a P-glycoprotein and CYP3A4 inhibitor, markedly raises the plasma concentrations and enhances the renin-inhibiting effect of aliskiren. Journal of clinical pharmacology 51(3):359-67 doi:10.1177/0091270010365885

Theunissen PT, Robinson JF, Pennings JL, de Jong E, Claessen SM, Kleinjans JC, et al. (2012) Transcriptomic concentration-response evaluation of valproic acid, cyproconazole, and hexaconazole in the neural embryonic stem cell test (ESTn). Toxicological sciences : an official journal of the Society of Toxicology 125(2):430-8 doi:10.1093/toxsci/kfr293

US EPA (2006). Re-registration eligibility decision for triadimefon and triadimenol and tolerance reassessment for triadimenol. Washington D.C.: Office of Pesticide Programs, United States Environmental Protection Agency.

US EPA (2008). Toxicological review of 2,2',4,4',5-pentabromodiphenyl ether (BDE-99). Washington D.C.: U.S. Environmental Protection Agency

Vachiery JL, Huez S, Gillies H, Layton G, Hayashi N, Gao X, et al. (2011) Safety, tolerability and pharmacokinetics of an intravenous bolus of sildenafil in patients with pulmonary arterial hypertension. British journal of clinical pharmacology 71(2):289-92 doi:10.1111/j.1365-2125.2010.03831.x

Vahidnia A, Romijn F, van der Voet GB, de Wolff FA (2008) Arsenic-induced neurotoxicity in relation to toxicokinetics: effects on sciatic nerve proteins. Chemico-biological interactions 176(2-3):188-95 doi:10.1016/j.cbi.2008.07.001

Vahidnia A, van der Straaten RJ, Romijn F, van Pelt J, van der Voet GB, de Wolff FA (2007) Arsenic metabolites affect expression of the neurofilament and tau genes: an in-vitro study into the mechanism of arsenic neurotoxicity. Toxicology in vitro : an international journal published in association with BIBRA 21(6):1104-12 doi:10.1016/j.tiv.2007.04.007

Vaidyanathan S, Jarugula V, Dieterich HA, Howard D, Dole WP (2008) Clinical pharmacokinetics and pharmacodynamics of aliskiren. Clinical pharmacokinetics 47(8):515-31

Veronese ME, McLean S, Hendriks R (1988) Plasma protein binding of amiodarone in a patient population: measurement by erythrocyte partitioning and a novel glass-binding method. British journal of clinical pharmacology 26(6):721-31

Viberg H, Fredriksson A, Eriksson P (2005) Deranged spontaneous behaviour and decrease in cholinergic muscarinic receptors in hippocampus in the adult rat, after neonatal exposure to

the brominated flame-retardant, 2,2',4,4',5-pentabromodiphenyl ether (PBDE 99). Environmental toxicology and pharmacology 20(2):283-8 doi:10.1016/j.etap.2005.02.004
Welsch F (2005) The mechanism of ethylene glycol ether reproductive and developmental toxicity and evidence for adverse effects in humans. Toxicology letters 156(1):13-28 doi:10.1016/j.toxlet.2003.08.010
Xuereb F, Chaignepain S, Breilh D, Godde F, Saux MC, Lenz C, et al. (2011) Quantitative analysis of erythropoietin in human plasma by tandem mass spectrometry. Analytical and bioanalytical chemistry 400(7):2073-84 doi:10.1007/s00216-011-4897-3
Yamada I, Suzuki F, Kamiya N, Aoki K, Sakurai Y, Kano M, et al. (2012) Safety, pharmacokinetics and resistant variants of telaprevir alone for 12 weeks in hepatitis C virus genotype 1b infection. Journal of viral hepatitis 19(2):e112-9 doi:10.1111/j.1365-2893.2011.01514.x
Yeung PK, Hubbard JW, Korchinski ED, Midha KK (1993) Pharmacokinetics of chlorpromazine and key metabolites. European journal of clinical pharmacology 45(6):563-9
Yung WK, Prados M, Levin VA, Fetell MR, Bennett J, Mahaley MS, et al. (1991) Intravenous recombinant interferon beta in patients with recurrent malignant gliomas: a phase I/II study. Journal of clinical oncology : official journal of the American Society of Clinical Oncology 9(11):1945-9
Zhang X, Cornelis R, de Kimpe J, Mees L, Lameire N (1998) Study of arsenic-protein binding in serum of patients on continuous ambulatory peritoneal dialysis. Clinical chemistry 44(1):141-7
Zimmer B, Lee G, Balmer NV, Meganathan K, Sachinidis A, Studer L, et al. (2012) Evaluation of developmental toxicants and signaling pathways in a functional test based on the migration of human neural crest cells. Environmental health perspectives 120(8):1116-22 doi:10.1289/ehp.1104489
